# Grow well/Crecer bien: a protocol for research on infant feeding practices in low-income families

**DOI:** 10.1186/s12889-020-09471-1

**Published:** 2020-09-21

**Authors:** Ann M. Cheney, Tanya Nieri, Ana Ramirez Zarate, Gretel Garcia, Lucero Vaca, Esmirna Valencia, Colleen Versteeg, Arlene Molina, Michael Castillo, Alison Tovar

**Affiliations:** 1 Department of Social Medicine Population and Public Health, 900 University Ave, Riverside, 92501 USA; 2 Department of Sociology, University of California Riverside, Riverside, USA; 3 School of Public Policy, University of California Riverside, Riverside, USA; 4 Graduate School of Education, University of California Riverside, Riverside, USA; 5 Riverside County Office of Education, Riverside, USA; 6 Orange County Head Start, Inc., Santa Ana, USA; 7 San Bernardino County Preschool Services Department, San Bernardino, USA; 8 Imperial County Office of Education, El Centro, USA; 9 Department of Nutrition and Food Sciences, University of Rhode Island, Kingston, USA

**Keywords:** Obesity, Infant feeding practices, Infant feeding styles, Low-income families, Nutrition education

## Abstract

**Background:**

The prevalence of obesity among children remains high. Given obesity’s significant lifelong consequences, there is great interest in preventing obesity early in life. There is a need to better understand the relation of common infant feeding styles and practices to obesity in infants using longitudinal study designs. There is also an urgent need to understand the role of caregivers other than mothers in feeding. A better understanding of variation in feeding styles and practices can inform the identification of risk groups and the tailoring of interventions to them.

**Methods:**

In partnership with Early Head Start programs across four counties in southern California, mothers and infants will be enrolled in a two-year longitudinal study collecting survey and anthropometric data. A subsample of mothers and their selected other caregivers will participate in qualitative research involving feeding diaries and dyadic interviews. The results will be used to develop and test an enhanced nutrition education program.

**Discussion:**

We outline a study methodology to examine feeding styles and practices and their association with early childhood obesity risk and enhance an existing intervention to promote healthy infant feeding and growth among children in low-income families.

## Background

Obesity prevalence among infants, toddlers, and preschool children in the United States (US) has doubled from the 1970s to the twenty-first century [1, 2]. Although rates have generally stabilized among some populations, racial/ethnic and socioeconomic disparities persist [3, 4]. For example, 9.4% of Latinx infants have a weight-for-length > 95th percentile relative to 6.6% of non-Latinx white infants. Although there has been a growing literature to better understand risk factors for obesity early in life, it remains unclear how feeding impacts early life risk for obesity among racial/ethnic minority children [5–9].

The American Academy of Pediatrics recommends exclusive breastfeeding as the optimal form of infant nutrition for the first 6 months and continued supplemental breastfeeding for 1 year. At 6 months, they recommend the introduction of a wide variety of nutrient-dense complementary foods. Starting at 9 months, they recommend three nutrient-dense meals and two or three small nutrient-dense snacks per day. Despite these guidelines racial/ethnic minority infants consume too many energy-dense foods and insufficient fruits and vegetables. Nationally representative research showed non-Latinx Black and Latinx infants between 6 and 11 months had lower intakes of fruits and vegetables and greater intakes of sweet and salty snacks compared to non-Latinx White infants [10, 11]. Research consistently shows common feeding practices deviate from these guidelines and are potential risk factors for early childhood obesity and targets for intervention: formula feeding, [12–16] combination feeding (breastmilk and formula), and the early introduction of solid foods [17, 18]. Other practices, such as early introduction of sugar sweetened beverages, are associated with obesity among older children and adults, [19] but their relation to obesity in infants is less explored [20, 21]. Research documents racial/ethnic disparities in these and related practices, such as feeding sugary drinks and “baby” cookies, [22] directing baby to empty the bottle, or propping the bottle [19, 23]. Such research offers insight into what infants are eating, but tells us little about how infants are fed.

Infant feeding styles and practices comprise the attitudes and behaviors that characterize caregivers’ approaches to maintaining or modifying children’s eating behaviors. We have limited information associating these feeding styles and practices with early childhood obesity [20, 21]. Responsive feeding, which involves properly interpreting and responding to the infant’s signals, is the recommended style [24]. Ethnic minority caregivers are more likely to exhibit non-responsive feeding styles (e.g., pressure feeding) relative to non-Latinx whites [6]. This difference in styles may be contributing to observed early-life obesity disparities [6]. Additionally, low levels of acculturation to the dominant American culture may be related to non-responsive styles and practices, [20, 25, 26] but results are inconsistent [27]. A better understanding of variation in feeding styles and practices can inform the identification of risk groups and the tailoring of interventions to them, as well as examining these relationships over time can help draw causal inferences and associations [28, 29].

Other caregivers (e.g., grandparents) are commonly involved in infant care and feeding, especially in ethnic minority families [30]. Non-parent caregivers’ infant feeding behaviors, especially those of relatives, have been associated with early childhood obesity [31–34]. However, there are only a handful of existing obesity interventions for young children that incorporate caregiver feeding styles and practices with nutrition advice, [35–37] and even fewer that account for the involvement of multiple caregivers in infant feeding [38]. They also start too late. Most begin with school-age children and target children 6 to 12 years of age [39]. The first 1000 days of life (i.e., conception through 24 months) constitute a critical period for optimal nutrition and development. Obesity interventions during this time period especially for low-income ethnic minority children, are still lacking [40–44]. Furthermore, few studies have engaged federally-funded programs, such as Early Head Start (EHS), which provides child development and family support services to infant and toddlers under the age of three in low-income families across the US [45].

To address these gaps, we developed the Grow Well/Crecer Bien project, a five-year community based participatory research (CBPR) study that is engaging EHS program leadership, staff, and families in the enhancement of existing nutrition education aimed to promote healthy infant feeding practices in the context of low-income families. The research aims to: 1) examine infant feeding styles and practices and their relation to infant growth and obesity in low-income families, 2) examine the role of other caregivers in infant feeding, and 3) develop and test an enhanced intervention, based on the information gained from the first two aims, that will address parents’ and other caregiver’s feeding styles and practices.

In this study we build on the Family Ecological Model to examine the social-ecological context that shapes infant feeding and child health, access to nutrition education, and the application of nutrition education to family systems [46]. This model provides structure when examining how factors in multiple systems operate together to shape child health. The microsystem includes the infant and their family. This unit is nested within the mesosystem that is the caregiving context (all the people who care for an infant). It may include a diversity of child caregiving knowledge and experience and a diversity of cultural values and norms shaping child care behavior. At the macrosystem level are public programs such as those serving low-income families. These programs influence the caregiving arrangements families make for their children and influence child health by shaping infants’ eating and, potentially, caregivers’ feeding. By viewing infant feeding in its ecological context, we can better understand how nutrition education translates to practice, affecting infant growth and obesity, and how the context could be better supported to improve child health. Furthermore, by incorporating multiple caregiver infant feeding into an intervention, we better address the diversity of U.S. families in terms of their caregiving context, reducing disparities using culturally appropriate programming.

The ultimate goal of this study is to reduce disparities in childhood obesity by incorporating healthy feeding styles and practices and multiple caregivers’ perspectives into existing nutrition education programs for low-income families. Our preliminary research with low-income Latina mothers in EHS identified the need for research to better understand and address the role of other caregivers. We showed that multiple caregivers, especially grandmothers, are involved in Latinx infant feeding. Although mothers understand the importance of healthy feeding, they may struggle to implement their knowledge in multiple-caregiver infant feeding contexts, increasing the risk for unhealthy infant growth and early childhood obesity [47]. This study addresses these latter concerns first by conducting a longitudinal study to characterize the caregiver context of infant feeding in low-income families and then by developing and testing a version of EHS nutrition programs enhanced by the incorporation of content addressing healthy feeding styles and practices with multiple caregivers.

## Methods

### Project overview

**Fig. 1:**
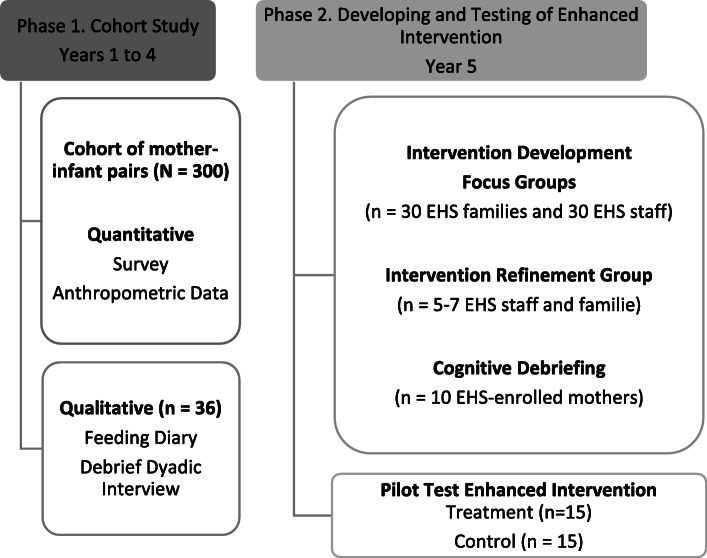
Overview of the two study phases

We use principles of CBPR, a collaborative approach combining the strengths of diverse partners and including all equitably in the research partnership [48]. In line with this approach, we will convene a community advisory board that meets quarterly, oversees the research, and approves all steps of the project. The research includes two phases. Figure 1 provides an overview of each phase of the research.

Phase 1 is a mixed-method longitudinal study intended to characterize the caregiver context of infant feeding in low-income families. We hypothesize that non-responsive feeding styles and non-recommended feeding practices will predict steeper infant growth trajectories and obesity status. To test this hypothesis, we will create a cohort of 300 mother-infant pairs to assess variation in mothers’ feeding styles and practices and model the relation of feeding styles and practices to infant growth from 2 to 24 months and obesity at 24 months. We will also obtain feeding data and in-depth information from a subsample of 36 mothers and their identified other caregivers to provide contextual data on other caregivers’ involvement in infant feeding.

Phase 2 will utilize qualitative methods to develop content to be added to EHS’ existing nutrition program to create the enhanced intervention. Approximately 30 EHS-enrolled families and 30 EHS staff will participate in focus groups. This phase also involves a pilot cluster randomized controlled trial comparing the enhanced program to the existing program. Thirty EHS-enrolled mothers will participate in the trial.

Before we started the research, the University of California Riverside Institutional Review Board (IRB) approved all data collection procedures, study instruments, and informed consent documents. Additionally, the study team and EHS programs completed Memoranda of Understanding outlining roles and responsibilities in the partnership.

### Sites and partnerships

The study is being conducted in partnership with EHS programs. The federally funded EHS program helps low-income mothers of children aged 0 to 3 years support the healthy development of their children through home- or site-based education and support services. EHS provides nutrition education through classes, opportunities to meet with nutritionists, and referrals to community nutrition resources. The classes follow the My Healthy Plate guidelines aligned with the U.S. Department of Agriculture’s healthy eating practice campaign (www.choosemyplate.gov), the cornerstone of government-sponsored nutrition programs for low-income mothers and children (https://wicworks.fns.usda.gov/nutrition-education). The curricula encourage portion control, food diversity, and nutrition content. Classes also include information on parental roles and responsibilities for food preparation and family meal time.

**Fig. 2:**
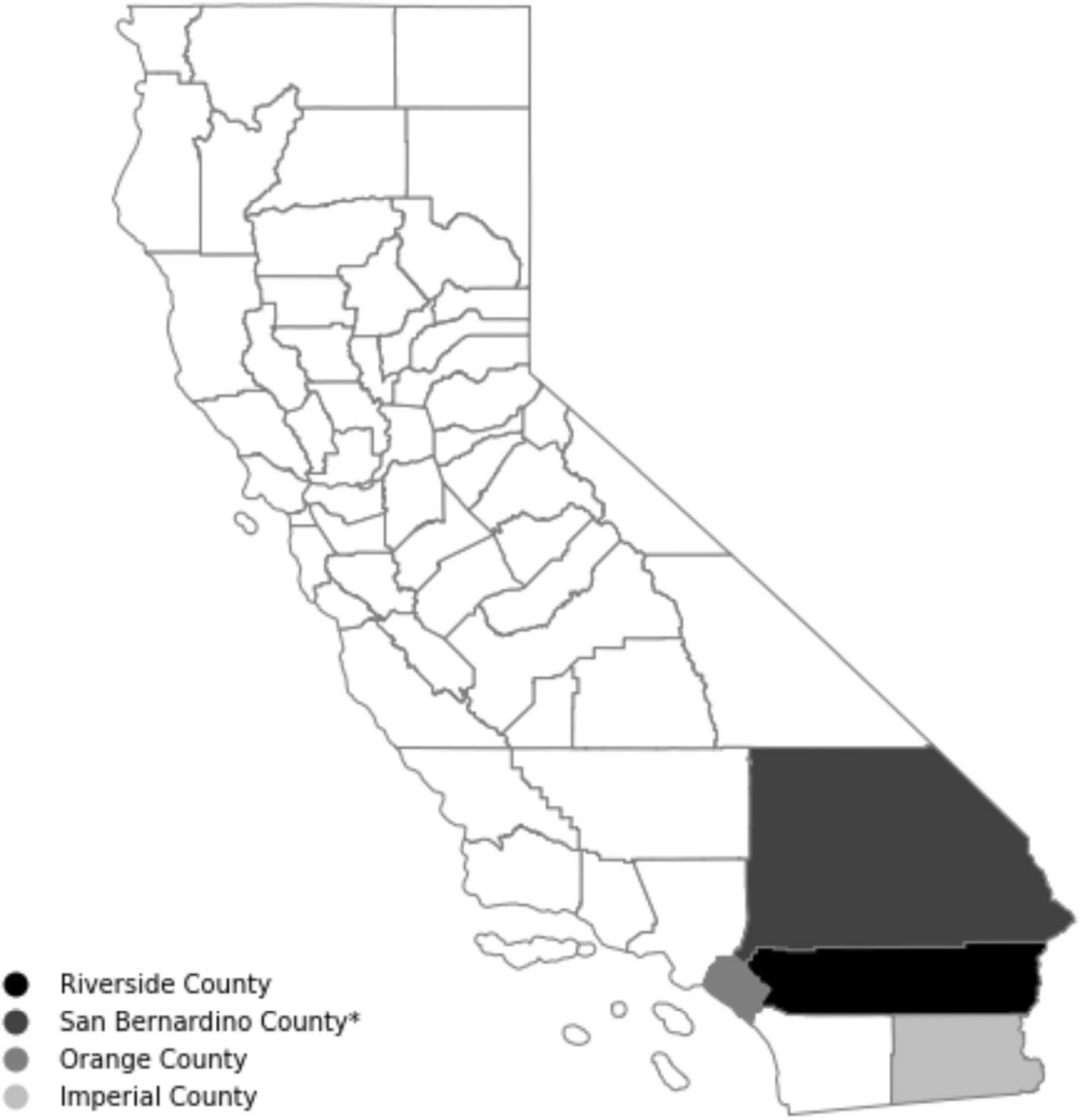
Location of Early Head Start Partners

The study involves partnerships with EHS programs across four bordering counties in southern California: Imperial, Orange, Riverside, and San Bernardino (see Fig. 2). These counties are characterized by often-overlapping pockets of high poverty and ethnic minority populations. Latinxs constitute the largest minority group in these counties -- 84.6% in Imperial, [49] 34.2% in Orange, [50] 49.6% in Riverside, [51] and 54% in San Bernardino [52] – with the largest subgroup, by far, being Mexicans. African Americans constitute the next largest minority group in Imperial (3.4%) [49], Riverside (7.2%) [51] and San Bernardino counties (9.4%) [52]; and the third largest minority group in Orange county (2.1%) [50]. While 12.8% of California’s population lives in poverty, [53] the poverty rates in three of these counties exceed the state average. A fifth (21.4%) of all persons live in poverty in Imperial [49], 12.7% in Riverside, [50] and 14.9% in San Bernardino [52]. Childhood obesity rates are likewise exceedingly high. Nationwide, 17% of children aged 2 to 19 years are obese [3]. Among California fifth graders, 49% of children in Imperial, 36% in Orange, 40% in Riverside, and 42% in San Bernardino counties are obese [54].

### Phase 1 - longitudinal study

During in-person visits when infants are 2, 6, and 12 months of age, trained study team members will administer^1^ individual face-to-face surveys with mothers (1.5 to 2 h) and collect anthropometric data from mother and child. During a 30-min visit at 24 months, anthropometric data only will be collected. Data will be collected as close to the infant’s target age as possible within a 3-month window, while facilitating maximal coordination with EHS programming and accommodation of families’ schedules. Visits will be part of regularly scheduled EHS visits and will take place either at the mother’s home or an EHS center. EHS staff will be present during data collection. Survey and anthropometric data will be recorded on laptops in Qualtrics, a web-based data collection software program. A subsample of 36 mothers from the cohort and 36 trusted other caregivers identified by these mothers will participate in feeding diary data collection when the infants are 2 and 6 months old and a dyadic interview when the infants are 6 months old.

#### Eligibility and recruitment

EHS staff, specifically home-based educators and center-based teachers, will facilitate the recruitment of mothers into the study. These staff members have direct contact with mothers either through weekly home-based visits or center-based daycare services. Prior to the start of recruitment, the study team held trainings with EHS staff in the four counties to provide an orientation to the study, including an overview of CBPR and the academic team’s partnership with EHS, the research aims, and recruitment procedures. During the training, home-based educators and center-based teachers along with EHS leadership decided on aspects of the recruitment procedures. Specifically, the discussion determined how to communicate among EHS staff/ supervisors and the study team when participants were identified.

To create a cohort of 300 mothers and their infants, EHS staff will distribute study flyers with eligibility criteria and study contact information to mothers on their caseload who are pregnant or who have an infant between the ages of 0 to 2 months. When mothers express interest, EHS staff will share the name and age of the child with the study team. Eligible mothers and their infants are enrolled in the study. Mothers must meet the following eligibility criteria: 1) be biologically female, 2) be 18 years of age or older, 3) have a child between ages 0 to 2 months that was born as a singleton, had a normal birth weight (greater than or equal to 5 lbs., 8 oz.), and be enrolled in EHS, 4) speak English or Spanish, and 5) be able to participate in four interviews over 2 years.

To recruit mothers and trusted other caregivers into the qualitative subsample, we will use purposive (non-random) sampling to identify mothers who reflect the sociocultural distribution of the quantitative cohort in terms of ethnicity and linguistic acculturation. We anticipate three groups within the subsample: English-speaking Latinas, Spanish-speaking Latinas, and mothers of other ethnicities. We expect that 12 mothers per group will achieve data saturation [55]. At the 2-month quantitative data collection visit, we will ask mothers if they consent to provide qualitative data in the form of: a) feeding diaries using the Baby Connect App when the infant is 2 and 6 months of age, the latter time point being associated with the introduction of solid foods, and b) a dyadic interview with the mother and trusted caregiver when the infant is 6 months. We will select 36 mothers on a rolling basis. Each mother must identify a trusted other caregiver to the focal infant to participate in a dyadic interview. Other caregiver inclusion criteria follows the Nurture Observational Study that examined multiple caregiver feeding effects on infant growth among a sample of minority (primarily African American) mother-child dyads [38]. A caregiver is any adult regularly involved in at least 3 h or more of infant care per week. This includes fathers, other partners, or non-parental caregivers (e.g., other family members, neighbors, babysitters, or childcare providers).

#### Survey measures

Infant weight and length will be measured to produce the two dependent variables: infant growth and obesity. Weight will be measured to the nearest 0.1 kg using calibrated Seca scales. Recumbent length will be measured to the nearest 0.5 cm using standard length rods. We will use the Centers for Disease Control (CDC) and Prevention SAS Macros for the World Health Organization (WHO) growth charts to convert weight and length measures into z-scores. WHO reference standards of gender-specific weight-for-length z-scores at ages 2, 6, 12, and 24 months will be used to evaluate infant growth and obesity.

Infant growth will be calculated as the growth trajectory over the first 24 months of life, using growth curve models of weight-for-length z-scores. We will explore the trajectory with different starting points, using the infant’s birth weight-for-length (reported by mother and converted to z-score) as baseline z-scores.

Obesity at 24 months will be calculated by the child’s body mass index (BMI), the standard clinical measure of obesity in children 2 years and older. We will assess this measure as kilograms of weight per square meter of length, and derive age- and gender-specific BMI percentiles, weight-for-age and BMI-for-age z-scores according to US national standards.

Feeding styles will be measured by the Infant Feeding Style Questionnaire (IFSQ), [56] which has 63 items on infant feeding beliefs and behaviors and 20 additional behavioral items related to solid feeding for children over 6 months of age. The questionnaire captures five feeding styles (i.e., laissez-faire, pressuring, restrictive, responsive, and indulgent) previously validated in a low-income sample of parents of infants and toddlers. Feeding practices will be measured by the 28 items (33 at 12 months) from the Baby Basic Needs Questionnaire (BBNQ) [57] pertaining to feeding, such as whether the child is breastfed exclusively or in combination with formula, when breastfeeding ended (if applicable), whether the child is exclusively formula fed, whether the child is fed solid foods, when solid foods were introduced, and what solid foods and other beverages are fed.

Covariates will be included in analyses as appropriate. At the 2-month visit, we will obtain infants’ birth weight and length, gestational age, and gender. We will also obtain mothers’ pre-pregnancy weight and height, gestational weight gain, age, level of education, race, ethnicity, acculturation (via the 42-item Abbreviated Multidimensional Acculturation Scale) [58], and levels of depression [59] and anxiety [60]. We will also obtain information about the household: zip code, highest level of education, total income, receipt of benefits, food insecurity, density (the number of people living in the home and the square footage of the residence), home ownership, childcare, and level of household chaos. At the 12-month visit, we will also measure mothers’ height and weight (see Additional file 1 for survey).

#### Statistical analysis

The study will use feeding styles and practices as two-way treatments, resulting in nested mixed models. In such models the fixed effects will be feeding styles (treated as a categorical variable with 5 levels for 5 feeding styles) and practices (treated as factors of composite scores for each feeding practice, such as exclusive breastfeeding and the introduction of solid food). The random effects will be caused by two types of correlations: those due to the infant characteristics, such as weight, and those due to clustering at multiple time points and within sites (infants’ families educated at the same sites). Accordingly, the R squared of the mixed model is expected to be at least 60%. The overall significance level α for a two-sided test is 0.05, and power 80%. Due to item similarities in a construct, the proposed significance level must be adjusted by the Bonferroni correction for multiple comparisons. In this study, the smallest adjusted significance level will be 0.0008 for each item in the construct of feeding beliefs and behaviors (0.05/63 items used in the construct of feeding practices). In addition, the hazard ratio across time points will be 1 for any reference level or group. Under these predefined conditions, the sample size will be 292, which was calculated in G*power 3.1.9.2.

Multiple imputation will be used if more than 5% of data is missing despite our efforts to prevent missing data. We will consider two types of effects: fixed and random. Thus, generalized linear mixed models will be ideal to analyze the data. Feeding styles and practices will be two categorical variables with fixed effects on the growth z score and obesity. Random effects will result from the nested grouping structure of infant families clustering in the same sites (like children in school classes) and the correlation between the variables, such as infant weight, collected from the same subjects across four time points. We will use generalized linear mixed models to examine if and how feeding styles and practices are associated with infant growth and obesity over time, and we will adjust these models for important covariates (e.g., mother’s pregnancy weight and age). Strongly associated items will be kept; all others will be excluded from further data analysis.

Generalized linear mixed models will be built to assess how the different constructs and/or sub-groups of feeding styles and practices relate to infant growth and obesity, adjusting for important covariates. We will employ Poisson regression to predict obesity, measured as the count of infants who became obese. The effect sizes for infant growth will be presented as how their weights change the score mean and/or summations of different constructs and their subgroups over time. The effect sizes for obesity will be presented as hazard ratios to evaluate comparative contributions of the construct and/or different sub-groups to obesity over time. Results of these analyses will be: 1) clustering of feeding style and practice items into subconstructs and subgroups which can be identified as meaningful clusters; 2) variation in feeding styles and practices within the sample; and 3) as whole latent variables, how these constructs or their subscales comparatively affect infant growth and obesity. This information will inform the development of the enhanced intervention components.

#### Feeding data

Qualitative data on infant feeding and other caregiver involvement in infant feeding will be collected using the Baby Connect application (app) (https://www.baby-connect.com/home) and diary debriefing interviews. The Baby Connect app is the most comprehensive baby tracking application on mobile application stores. This app also has a web interface for parents who do not have an iPhone or Android smartphone to view and enter information. If participants do not have a smartphone or access to a computer with internet, we will provide them with a tablet with the app downloaded onto it.

Baby Connect is set up like a log to track solid food feeding, bottle feeding, and breastfeeding. Within each of these types, mothers can further select category options (e.g., Bottle feeding includes milk, water, formula, or juice). For each entry, the user can add text in a notes section. We will ask participants to describe other caregiver involvement in this section. The goal is to understand whether and how mothers translate their EHS nutrition education to practice in the context of other caregivers’ involvement in infant feeding. This app will provide data on: 1) whether mothers give directives to other caregivers, 2) if other caregivers follow those directives, and 3) how mothers respond to other caregivers’ infant feeding styles and practices.

Infant feeding data will focus on who fed the infant, how the infant was fed (e.g., breast or bottle fed, spoon fed), what food was fed (e.g., milk, thickeners, solid food), when/why the feeding ended, and interpersonal interactions during the feeding. This information will allow us to examine feeding styles and practices. Given that use of smartphones is prevalent among low-income populations, [61] we will use a popular app for data collection, in lieu of a paper-and-pencil diary, to reduce participant burden and increase compliance.

Previous research asked mothers to collect weekly diaries over 6 weeks and respond to six questionnaires, resulting in six data points and sufficient infant feeding data [62]. In consideration of participant retention, repeat assessment, and the daily demands of having an infant, we will collect six data points over 48 h, capturing at least two each of morning, afternoon, and evening feeding occasions. Data from the Baby Connect app will be downloaded to a .csv data format immediately following the end of the 48-h data collection period.

Within 3 days of completion of the feeding diary, trained interviewers will conduct 45–60 min feeding data de-briefings by phone with mothers. Interviewers will use a semi-structured interview guide to elicit in-depth data on feeding occasions identified via the feeding diary data related to conflict, such as differences in feeding styles and practices and communication approaches among caregivers (see Additional file 2 for interview guide).

#### Dyadic interviews

In-person dyadic interviews with mothers and other caregivers will be conducted after the completion of the feeding diary data collection when the infant is 6 months of age. Dyadic interviews, an interactive technique involving an interviewer and two participants, [63] will encourage the mother and other caregiver to interact with each other in response to open-ended questions about feeding events documented in the feeding diary. This technique allows the comments of one participant to evoke responses from the other participant, provides interviewers with an understanding of the relationship dynamic, and allows participants to co-construct their understanding of past events within their personal relationship [64]. A semi-structured interview guide will be used to elicit information on: 1) similarities and differences in infant feeding styles and practices between caregivers, 2) sources of conflict in infant feeding practices, and 3) ways to overcome conflict or communicate about disagreements (see Additional file 3 for interview guide). At the end of the dyadic interview, the trusted other caregivers will complete a brief survey to collect basic demographic information and responses to the IFSQ [56, 65].

#### Qualitative analysis of feeding data

The feeding data from the Baby Connect app and transcripts of the debriefing interviews will be analyzed using a deductive approach. A priori or predetermined codes from the interview guide (e.g., disagreement/conflict) and existing literature on common feeding styles and practices will be used as initial codes, as will key terms identified during the debriefing (e.g., communication approaches). We will use word-based techniques (e.g., Keywords-in-Context), which are rapid and efficient ways to locate specific examples of text for theme development. For feeding data, we will use this analytic technique to identify text related to caregiver roles, differences, and conflicts in infant feeding, which will inform probes for subsequent dyadic interviews.

The dyadic interviews will be transcribed and the textual data will be analyzed using an inductive approach. The analysis will begin during data collection to ensure theoretical saturation [66]. Text, mother and caregiver demographics, and IFSQ data will be imported into MAXQDA, [67] a qualitative, mixed-methods data analysis software program. Following MacQueen et al., [68] we will use open coding to read texts line by line to identify emergent themes and develop a team-based codebook. Once the codebook is established, in-vivo coding will be used to independently apply the codes to the same transcripts to assess inter-coder agreement, repeating this process until an acceptable inter-coder reliability of .80 is reached. Once an agreement is established, the analysts will apply the codes to the textual data. Axial coding, or constant comparison, will then be used to conduct a comparative analysis among mothers and trusted caregivers by their IFSQ scores.

#### Tracking and retention

At the 2-month visit, researchers will explain to participants the importance of maintaining up-to-date contact information, provide a prepaid mailer with a change of address/phone number form, and ask for names and contact information for three additional adults (e.g., nearby relatives, friends) who may be contacted in the event participants become difficult to reach over the course of the study. Participants will receive the study team’s phone, email, and web contact information to communicate any scheduling changes or changes in contact information. To maximize participant retention, we will use our partnership with EHS to track participants who enroll in the study through EHS records, including home addresses and forwarding addresses if families relocate. To minimize travel or other burden on study participants, we will align in-person data collection procedures with the regular EHS site or home visit for EHS participants. As incentives for participation in the survey, participants will receive $30 for visit 1, $40 for visit 2, $50 for visit 3, and $60 for visit 4, the amount increasing over time to encourage continued participation. Prior obesity trials with children had an average retention rate of 86% [69]. Feeding data participants will receive $50 each provision of feeding data. Each dyadic interview participant will receive $30.

### Phase 2 - enhanced intervention development and pilot testing

This phase involves two steps: developing intervention components that can be added to EHS’ existing nutrition education program and testing the enhanced intervention.

#### Step 1: intervention development

To develop the intervention components on feeding styles and practices and multiple caregivers, we will engage in a process of information gathering, focus groups, intervention refinement, and cognitive debriefing interviews. We will identify existing evidence-based interventions and “best practices” from the literature on nutrition, early childhood obesity prevention, and culturally grounded interventions for low-income families that inform intervention development. The study team will gather information on infant feeding and childhood obesity interventions, such as the Greenlight NOURISH Project, [70] the Soothe/Sleep project, [71] and projects in the Arikpo et al. [72] systematic review of randomized controlled trials of infant feeding interventions. It will synthesize this information, along with any relevant information from the current EHS nutrition education program and Phase 1 study findings. Because our pilot work revealed many EHS-enrolled mothers were also enrolled in the Special Supplemental Nutrition Program for Women, Infants, and Children (WIC), [47] we will also synthesize relevant WIC nutrition education material to ensure intervention components are consistent with WIC messages.

Once we develop drafts of the intervention components, we will conduct 6 focus groups of 10 people each (3 with mothers and other caregivers, 3 with EHS staff) to gather feedback and any additional ideas on what should be included. For nonprobability samples, 80% of themes can be identified within two to three focus groups and 90% within three to six focus groups [73]. The study team will facilitate the groups and take notes to document key discussion points and themes. We will ask for participant feedback on EHS’s current nutrition education, relevant findings from Phase 1, and components of existing interventions relevant to infant feeding styles and practices and multiple caregiver feeding. We will prompt discussion about the material and how each may fit with the needs of low-income families, encouraging participants to suggest other optimal intervention components for further development. We will also ask about infant feeding: challenges in multiple caregiver contexts, personal and environmental facilitators and barriers to infant feeding, and motivators for improving infant feeding (see Additional file 4 for interview guide). Focus groups are ideal as they allow participants to build on each other’s ideas [74].

Focus groups will be audio-recorded and analyzed using template and matrix analysis, a rapid analytic technique [75]. The study team will listen to audio recordings and insert data, including illustrative excerpts from the interviews, in the templates. Next, a matrix (focus group × domain) will be created, and data from each template will be inserted into the matrix. This approach facilitates identifying patterns across groups. We will create two separate matrices (one from groups with mothers and caregivers and one from groups with EHS staff) from which we will create two separate lists of identified content for the intervention components. The Community Advisory Board will review the content identified for the components and make recommendations. Ultimately, the study team will use the recommendations to develop the components.

After the focus groups, intervention refinement working group meetings and cognitive debriefing interviews will be held to finalize the intervention components. We will create an intervention refinement working group of 5–7 people: academics, EHS-enrolled mothers, and EHS staff. Findings from Phase 1 and the focus groups will help inform the intervention components; however, we expect to develop at least two intervention components, both of which may be characterized by educational sessions (60–90 min each), text messages, and printed materials. The working group will develop the intervention components and suggest refinements to them, which will then be incorporated in an iterative fashion and brought back to the group, [76] which may need to meet 2–3 times to revise the components. The study team will incorporate any suggested changes. Before finalizing the intervention components, they will be translated from English to Spanish and back, ensuring both versions are culturally relevant, accessible, and available.

We will then conduct one-time cognitive debriefing interviews with 10 EHS-enrolled mothers. We will interview 3 mothers in English and 3 in Spanish and incorporate their feedback. After revisions are incorporated, we will interview two other mothers in English and 2 in Spanish. In previous work, we found that feedback from 3 to 5 interviews (in any one language) is sufficient. We will interview in the two language groups because components will be in both English and Spanish. In the interview, participants will review the materials for the components, followed by relatively structured questions to assess understanding of the material. The study team will conduct interviews, eliciting information on: 1) appropriateness of language, 2) relevance of material, and 3) unclear, confusing, or misunderstood material [77]. The 75-min, in-person interviews will occur at EHS sites and be audio-recorded. The interviewer will also take notes. Immediately following the interview, the interviewer will listen to the audio recording and use their notes to identify needed revisions [78]. These notes will be compiled, discussed by the study team and CAB members, and incorporated. The results for Step 1 will be validation-ready drafts of the components that will comprise the enhanced intervention.

#### Step 2: pilot testing via a cluster randomized controlled trial (RCT)

To pilot test the enhanced intervention at EHS sites, we will compare the enhanced intervention’s feasibility, acceptability, and preliminary efficacy in improving maternal feeding styles and practices to EHS’ existing intervention. Prior to designing a larger trial, it is imperative to have data on recruitment processes (% eligible, reasons for not participating, recruitment yield), baseline process data (% completion, time range from recruitment to intervention, attendance/sessions completed), baseline survey data, and outcome data including preliminary effect sizes. These data will inform the sample size for a future RCT.

With EHS staff, we will identify two EHS sites and optimal dates/times during their regular monthly site-based group meetings for intervention implementation. Sites will be randomly assigned, via coin toss, to the treatment or control condition. The enhanced intervention will be implemented in a two-month period to align with the implementation of existing nutrition education. We will train an EHS staff person at each treatment site to deliver the enhanced intervention. At the control sites, existing nutrition education will be provided as usual.

Random selection will be used in which 15 mothers from both sites will be selected to participate (30 mothers total, 15 treatment and 15 control). This sample is appropriate for a pilot whose aims are to assess feasibility, acceptability, and preliminary efficacy [79]. EHS staff will recruit EHS-enrolled mothers as part of their regular outreach with program families, using the eligibility criteria for Phase 1.

The research team will observe implementation for fidelity and qualitative debriefing interviews with five EHS staff members, including the implementers, to assess feasibility (e.g., fit with existing programming and program requirements, participant retention) and acceptability (e.g., format and length, cultural relevance). Members of the study team will attend the EHS group meetings to administer brief paper-and-pencil pre- and post-tests to participating mothers; these members will be blinded to the experimental control. At treatment sites pre-tests will be administered immediately prior to intervention implementation, and post-tests will be administered 1 month after the end of implementation. At control sites, the pre- and post-tests will be administered 3 months apart, in alignment with the treatment site data collection.

To determine preliminary efficacy, pre- and post-tests will assess increases in participants’ knowledge and use of responsive feeding styles and recommended feeding practices using the IFSQ and BBNQ; decreases in risky feeding practices using the BBNQ; increases in knowledge of the role of other caregiver feeding practices (based on results from Phase 1); and increases in use of recommended strategies for negotiating infant feeding with other caregivers (based on results from Phase 1 and Step 1 of Phase 2). Measures for the latter two outcomes will be developed by the research team. Post-tests of the treatment group will also assess the enhanced intervention’s acceptability (e.g., format and length), cultural relevance, and overall satisfaction (e.g., “In general, how satisfied were you with the intervention?”).

We will consider the intervention feasible if qualitative analysis of EHS staff feasibility data (observations and debrief interviews) reveals no major concerns and at least 80% of intervention participants are retained at the post-test. We will consider the intervention to be acceptable if at least 80% of participants indicate that they were “satisfied” or “very satisfied” with the intervention. With regard to preliminary efficacy, our goal for the pilot is to determine whether the enhanced intervention positively impacts the proximal outcomes of feeding styles and practices and caregiver context, with the idea that if it does, it will positively impact on the distal outcomes of infant growth and obesity as tested in a future trial. Using quantitative analysis, we will assess pre- to post-test changes in outcomes in the treatment and control conditions, using general linear mixed models and/or permutation tests. We will consider the intervention preliminarily efficacious if the changes trend in the desired direction. The results of Step 2 will be the enhanced intervention and information on the feasibility, acceptability and preliminary efficacy of the enhanced intervention.

#### Tracking and retention

To maximize retention, we will track participants through EHS records and align data collection with the regular EHS site visits. Focus group participants will receive $30. Working group participants will receive $75 for each meeting. Cognitive debriefing interview participants will receive $30. Pilot implementation participants will receive $25 at pretest and $35 at posttest. Pilot debriefing participants will receive $30.

## Discussion

Grow Well/Crecer Bien is a timely project, critical for addressing disparities in early childhood obesity. It innovatively moves beyond healthy eating to examine healthy feeding. In contrast with prior research, we conceptualize feeding as both a social and biological activity. Feeding provides an opportunity for people to fulfill social roles, transmits culture (e.g., consumption of culture-specific foods), and teaches behavioral norms [80, 81]. Therefore, we focus on the interactions between children and their caregivers, examining how children are fed (styles and practices) and why. Using the methods of feeding data debriefing and dyadic interviews, we focus on the interpersonal interactions between mothers and other caregivers as a contextual factor shaping infant feeding and, in turn, infant growth and obesity.

Our work acknowledges the role of other caregivers in infant feeding. US demographics have changed dramatically in recent years, [82] let alone since the development of nutrition interventions in the 1940s [83]. Existing interventions and programs tend to focus on single caregivers and do not formally incorporate other caregivers in nutrition education and programs [84, 85]. Furthermore, prior research on feeding focuses on the parents (usually mothers) in isolation, whereas this project focuses on mothers and trusted other caregivers. Using specific examples from the feeding data, collected through a well-known and user-friendly baby feeding app, and the dyadic interview data, we can understand who is involved in feeding, the roles and responsibilities of other caregivers in feeding, similarities and differences in feeding styles and practices among caregivers, and how feeding decisions are negotiated among caregivers. We will develop intervention components intended to enable mothers to better translate their nutrition education to practice in multiple caregiver contexts.

There is an urgent need to begin obesity prevention early in life [86, 87]. Despite EHS reaching a vulnerable and ethnically diverse population, few studies have incorporated obesity prevention with infants in this setting. There is an opportunity to integrate evidence-based feeding practices of the family system into EHS’ current programming. Rather than develop a new, separate intervention, we modify an existing intervention to translate to practice new knowledge about feeding in multiple caregiver contexts. Prevention scientists argue that building on existing interventions may be an ideal way to efficiently and cost-effectively address the need for prevention [88–90]. By using CBPR methods to modify an existing curriculum, we ensure that the enhanced intervention is feasible for EHS, the implementing organization, and acceptable to the target population.

We account for several potential limitations. Our research design involves building on Phase 1 findings. If unable to build on the findings, we will pull from the existing literature and relevant Phase 1 data to identify potential components for nutrition education and obesity prevention in early life to complete Phase 2. Additionally, other caregivers are currently not part of EHS programming. Were study participants to recommend an intervention component that includes other caregivers as participants in nutrition education, we could develop a component to include the participation of other caregivers as guests in an intervention session. This would enable other caregivers to receive education on feeding styles and practices between multiple caregivers. In this case we would modify the pilot test to also collect pre- and post-tests from other caregivers who receive the implemented component. It would be ideal to collect survey data from all trusted other caregivers as well as qualitative data from the full cohort. However, budgetary and time constraints limit our ability to carry out such data collection and analysis. Furthermore, Covid-19 has delayed the start of this project and recruitment and in-person data collection methods may need to be adjusted accordingly.

Despite these limitations, our project stands to make important contributions by generating new knowledge about feeding styles and practices in multiple caregiver contexts and their relation to infant growth and obesity while integrating nutrition and obesity knowledge to enhance intervention. It fulfills our EHS partners’ priority of identifying risk factors for infants and toddlers in low-income families. If the pilot RCT proves efficacious and feasible, our next step will be to conduct a large-scale RCT with the long-term goal of wide dissemination of the intervention and engagement of WIC and other government-sponsored nutrition programs in the implementation of enhanced nutrition education to prevent obesity in early life. Through better understanding of how infant feeding styles and practices and other caregivers shape infant growth and obesity, and effective interventions that incorporate this understanding, we stand to reduce the exposure of children and the nation’s health care system to significant preventable comorbidity.

## Supplementary information

**Additional file 1: Appendix A**. Longitudinal Survey.

**Additional file 2: Appendix B**. Feeding Diary Debriefing Interview.

**Additional file 3: Appendix C**. Dyadic Interview Guide.

**Additional file 4: Appendix D**. Focus Group Guide.

## Data Availability

The datasets used and/or analyzed during the current study are available from the corresponding author upon reasonable request.

## Abbreviations

BBNQ: Baby basic needs questionnaire
CBPR: Community based participatory research
EHS: Early head start
IFSQ: Infant feeding style questionnaire
RCT: Randomized controlled trial

## Footnotes

1 In light of the current global pandemic involving COVID-19, data collection may move from in-person to online.
