# Supplementary material for: Grow well/Crecer bien: a protocol for research on infant feeding practices in low-income families
Source: BMC Public Health. 2020 Sep 21;20:1431. doi: 10.1186/s12889-020-09471-1 (PMC7503435; doi:10.1186/s12889-020-09471-1)
Supplement: Supplementary file 1 — Additional file 1: Appendix A. Longitudinal Survey. [file 12889_2020_9471_MOESM1_ESM.pdf]

Appendix A. Aim 1a Wave 1 Survey English-Spanish

Participant Copy INFANT CHARACTERISTICS

We would like to begin by asking some questions about your baby, (Name of baby).

	1a. Month	1b. Day	1c. Year
What is your baby's birth date?			

	Boy	Girl	Other
2. What is your baby's gender?			

	Pounds	Ounces
3. What was your baby's weight at birth?		

	Inches
4. What was your baby's length at birth?	

	Gestational Age in weeks
5. Did you give birth to the child? At how many weeks did you give birth?	

	Yes	No
6. Does your baby attend daycare?		

	Daycare in a center	Daycare in a private home
7. What kind of daycare does your baby attend?		

CARACTERÍSTICAS INFANTILES

Nos gustaría comenzar haciendo algunas preguntas sobre su bebé, (Nombre del bebé).

	1a. Mes	1b. Día	1c. Año
¿Cuál es la fecha de nacimiento de su bebé?			

	Niño	Niña	Otro
2. ¿Cuál es el género de su bebé?			

	Libras	Onzas
3ab. ¿Cuál era el peso de su bebé al nacer?		

	Pulgadas
4. ¿Cuál fue la longitud de nacimiento del bebé?	

	Edad gestacional
5. ¿Usted dio a luz al niño? ¿A cuántas semanas dio a luz?	

	Sí	No
6. ¿Asiste su bebé a la guardería?		

	Guardería en un centro	Guardería en un hogar privado
7. ¿A qué tipo de guardería asiste su bebé?		

	Average hours/day
8. How many hours per day on average does your baby attend daycare?	

Aside from day care, does your baby spend any time in the care of any of the following people other than you? If yes, how many hours per day on average?	Average hours/day
9a. Baby's father	
9b. Your mother	
9c. Your mother in law	
9d. Other family member	
9e. Non-family member babysitter	
9f. Other person<br>9g. Please specify:	

	Average hours/day
10. How many hours per day on average does your baby sleep during the day?	
11. How many hours per day on average does your baby sleep during the night?	

	Yes	No
12. Is there a television in the room where your baby sleeps during the day?		
13. Is there a television in the room where your baby sleeps during the night?		

	Yes	No
14. Has your baby had antibiotics since birth?		

	Promedio de horas/día
8. ¿Cuántas horas por día, en promedio, atiende su bebé a guardería?	

Además de la guardería, ¿pasa su bebé algún tiempo al cuidado de alguno de los siguientes que no sea usted? Si sí, ¿cuántas horas por día, en promedio?	Promedio de hora/día
9a. El padre del bebé	
9b. Su madre	
9c. Su suegra	
9d. Otro miembro de su familia	
9e. Otra niñera quien no sea miembro de su familia	
9f. Otra persona<br>9g. Por favor especifique:	

	Promedio de horas/día
10. ¿Cuántas horas por día en promedio duerme su bebé durante el día?	
11. ¿Cuántas horas al día en promedio duerme su bebé durante la noche?	

	Sí	No
12. ¿Hay un televisor en la habitación donde duerme su bebé durante el día?		
13. ¿Hay un televisor en la habitación donde duerme su bebé durante la noche?		

	Sí	No
14. Su bebé ha tomado antibióticos desde que nació?		

	Number of hours	Number of minutes
15. On average, how many hours or minutes does your baby spend watching something on a screen, such as a TV, smart phone, tablet, etc., on a typical day?		

	Yes	No
16a. Is your baby covered by health insurance?		

	Private insurance	Public insurance (MediCal/Medicare)	Other. 16c. Please specify.
16b. What health insurance does your baby have?			

	IEHP	Molina	Cal Optima	California Health and Wellness	InnovAge PACE	SCAN Health Plan
16d. In which MediCal program is your child enrolled?						

	Número de horas	Número de minutos
15. En promedio, ¿cuántas horas o minutos pasa su bebé mirando algo en la pantalla, como un televisor, teléfono inteligente, tableta, etc., en un día típico?		

	Sí	No
16a. ¿Su bebé está cubierto por un seguro de salud?		

	Seguros privados	Seguros públicos (MediCal/Medicare)	Otros. 16c. Por favor especifique.
16b. ¿Qué seguro de salud tiene su bebé?			

	IEHP	Molina	Cal Optima	California Health and Wellness	InnovAge PACE	SCAN Health Plan
16d. En qué programa MediCal está inscrito su hijo?						

FEEDING STYLES AND PRACTICES

Now, let’s turn to the topic of feeding your baby.

The following questions ask for your opinion about infant feeding.

To what extent do you agree or disagree with the following statements?	Disagree	Slightly Disagree	Neutral	Slightly Agree	Agree
17a. It's okay to prop an infant's bottle					
17b. It’s important for an infant to finish all of the milk in his/her bottle					
17c. Cereal in the bottle will help an infant sleep through the night					
17d. Putting cereal in the bottle is good because it helps an infant feel full					
17e. An infant less than 6 months old needs more than formula or breastmilk to be full					
17f. An infant less than 6 months old needs more than formula or breastmilk to sleep through the night					
17g. The best way to make an infant stop crying is to feed him/her					
17h. When an infant cries, it usually means s/he needs to be fed					
17i. It is important that the parent decides how much an infant should eat					
17j. An infant should never eat fast food					
17k. (Name of child) knows when s/he is full					
17l. (Name of child) knows when s/he is hungry and needs to eat					

ESTILOS Y PRÁCTICAS DE ALIMENTACIÓN

Ahora, pasemos al tema de alimentar a su bebé**.**

Las siguientes preguntas se refieren a cómo piensa usted acerca de la alimentación infantil.

¿En qué medida está de acuerdo o en desacuerdo con las siguientes declaraciones?	Desacuerdo	Ligeramente en desacuerdo	Neutral	Ligeramente de acuerdo	Acuerdo
17a. Está bien dejar el biberón encima de algo					
17b. Es importante que el bebé termine toda la leche en su biberón					
17c. El cereal en el biberón ayuda al bebé que duerma toda la noche					
17d. Poner cereal en el biberón es bueno porque ayuda al bebé a sentirse lleno					
17e. Un bebé de menos de 6 meses de edad necesita más que fórmula o leche materna para estar lleno					
17f. Un bebé de menos de 6 meses de edad necesita más que fórmula o leche materna para dormir durante la noche					
17g. La mejor manera de hacer que el bebé deje de llorar es alimentarlo					
17h. Cuando el bebé llora, generalmente significa que necesita ser alimentado					
17i. Es importante que el padre decida cuánto debe comer un bebé					
17j. Un bebé nunca debe comer comida rápida					
17k. (Nombre del bebé) sabe cuándo está lleno/a					
17l. (Nombre del bebé) sabe cuando tiene hambre y necesita comer					

The following questions ask about how you feed your baby. I'm going to ask about the types of food you give your baby and how often you feed each type.

In the past seven days, how often was your baby fed each food listed? Include feedings by everyone who feeds the baby and include snacks and night-time feedings. If you give the baby that type of food each day, then you can tell me the number of feedings of that food per day. If you give the baby that type of food less than daily, you can tell me the number of feedings of that food per week.	Number of feedings per day	Number of feedings per week
18a. Breast milk		
18b. Formula		
18c. Cow's milk		
18d. Other milk: soy milk, rice milk, goat milk, etc.		
18e. Other dairy foods: yogurt, cheese, etc.		
18f. Other soy foods: tofu, frozen soy desserts, etc.		
18g. 100% fruit juice		
18h. 100% vegetable juice		
18i. Sweet drinks: juice drinks, soft drinks, sweet tea, hierbabuena tea, Kool-Aid, etc.		
18j. Baby cereal		
18k. Other cereals and starches: breakfast cereals, teething biscuits, crackers, breads, pasta, rice, etc.		
18l. Fruit, not including fruit juice or fruit in syrup		
18m. Vegetables, not including french fries		

Las siguientes preguntas se refieren a cómo alimenta a su bebé. Voy a preguntar sobre los tipos de alimentos que le da a su bebé y con qué frecuencia alimenta cada tipo.

En los <u>últimos siete días</u>, ¿con qué frecuencia se alimentó a su bebé con cada alimento que están en la tarjeta? Incluya la alimentación de todos los que alimentan al bebé e incluya aperitivos y comidas nocturnas. Si le da al bebé ese tipo de alimento todos los días, entonces puede decirme la cantidad de alimentos que ese alimento toma por día. Si le da al bebé ese tipo de alimento menos que diariamente, puede decirme la cantidad de alimentación de ese alimento por semana.	Número de alimentaciones por día	Número de alimentaciones por semana
18a. Leche materna		
18b. Leche de fórmula		
18c. Leche de vaca		
18d. Otra leche: leche de soya, leche de arroz, leche de cabra, etc.		
18e. Otros productos lácteos: yogur, queso, etc.		
18f. Otros alimentos de soya: tofu, postres de soya congelados, etc.		
18g. 100% jugo de fruta		
18h. 100% jugo de vegetales		
18i. Bebidas azucaradas: bebidas de jugo, refrescos, té dulce, hierbabuena, Kool-Aid, etc.		
18j. Cereales para bebés		
18k. Otros cereales y almidones: cereales para el desayuno, galletas para la dentición, galletas, panes, pastas, arroz, etc.		
18l. Frutas, sin incluir jugo de fruta o jarabe de fruta		
18m. Verduras, sin incluir papas fritas		

In the past seven days, how often was your baby fed each food listed? Include feedings by everyone who feeds the baby and include snacks and night-time feedings. If you give the baby that type of food each day, then you can tell me the number of feedings of that food per day. If you give the baby that type of food less than daily, you can tell me the number of feedings of that food per week.	Number of feedings per day	Number of feedings per week
18n. French Fries or other fried potatoes		
18o. Meat, chicken, combination dinners		
18p. Fish or shellfish		
18q. Peanut butter, other peanut foods, or nuts		
18r. Eggs		
18s. Sweet foods: candy, cookies, cake, etc.		
18t. Baby snacks such as melts or puffs		
18u. Squeezable baby food pouches		
18v. Other food.<br>18w. Please specify:		

	Never	Rarely	Half of the time	Most of the time	Always
19. How frequently does (Name) watch something on a screen (TV, tablet, phone, etc.) while eating?					

How frequently do you engage in the following behaviors?	Never	Rarely	Half of the time	Most of the time	Always
20. When your baby has a bottle, you prop it up					
21. You watch something on a screen (TV, tablet, phone, etc.) while feeding your baby					

En los <u>últimos siete días</u>, ¿con qué frecuencia se alimentó a su bebé con cada alimento que están en la tarjeta? Incluya la alimentación de todos los que alimentan al bebé e incluya aperitivos y comidas nocturnas. Si le da al bebé ese tipo de alimento todos los días, entonces puede decirme la cantidad de alimentos que ese alimento toma por día. Si le da al bebé ese tipo de alimento menos que diariamente, puede decirme la cantidad de alimentación de ese alimento por semana.	Número de alimentaciones por día	Número de alimentaciones por semana
18n. Papas fritas, u otros tipos de papas fritas		
18o. Carne, pollo, cenas combinadas		
18p. Pescado o mariscos		
18q. La crema de cacahuate, otros alimentos de cacahuate o nueces		
18r. Huevos		
18s. Alimentos dulces: dulces, galletas, pasteles, etc.		
18t. Aperitivos para bebés, como melts o puffs		
18u. Bolsitas exprimibles de comida		
18v. Otro alimento.<br>18w. Por favor especifique:		

	Nunca	Raramente	Mitad de las veces	La mayoría de las veces	Siempre
19. ¿Con qué frecuencia (Nombre) mira algo en una pantalla (TV, tableta, teléfono, etc.) mientras come?					

¿Con qué frecuencia usted participa en los siguientes comportamientos?	Nunca	Raramente	Mitad de las veces	La mayoría de las veces	Siempre
20. Cuando su bebé tiene un biberón usted lo sostiene hacia arriba con algo					
21. Usted ve algo en una pantalla (TV, tableta, teléfono, etc.) mientras alimenta a su bebé					

	Yes	No
22a. Have you ever breastfed your baby?		
22b. Are you currently breastfeeding this baby?		

	Days (if younger than 2 weeks)	Weeks
22c. How old was your baby when you completely stopped breastfeeding and pumping milk?		

	Yes	No
22d. While breastfeeding your baby, did you ever or do you use a screen, such as watching TV, using your smartphone, etc.?		

	Never while feeding	Rarely	Half of the time	Most of the time	Every time while feeding
22e. How frequently did you or do you use a screen while breastfeeding?					

How frequently do you engage in the following behaviors?	Never	Rarely	Half of the time	Most of the time	Always
23a. You keep track of what food (name of child) eats					
23b. You keep track of how much food (name of child) eats					
23c. You make sure (name of child) does not eat sugary food like candy, ice cream, cakes or cookies					
23d. You make sure (name of child) does not eat junk food like potato chips, Doritos and cheese puffs					

23e. You try to get (name of child) to finish his/her food					

	Sí	No
22a. ¿Alguna vez ha amamantado a su bebé?		
22b. ¿En este momento está amamantando a su bebé?		

	Días (si es menor de 2 semanas)	Semanas
22c. ¿Qué edad tenía su bebé cuando dejó de amamantar y extraer leche por completo?		

	Sí	No
22d. Mientras amamantaba a su bebé, ¿alguna vez o usó una pantalla, como mirar televisión, usar su teléfono inteligente, etc.?		

	Nunca mientras se alimenta	Raramente	Mitad del tiempo	Mayoría del tiempo	Cada vez que se alimenta
22e. ¿Con qué frecuencia usó o usa una pantalla durante la lactancia?					

¿Con qué frecuencia usted participa en los siguientes comportamientos?	Nunca	Raramente	Mitad de las veces	La mayoría de las veces	Siempre
23a. Usted registra de qué comida (nombre del bebé) come					
23b. Usted registra la cantidad de comida que come (nombre del bebé)					
23c. Usted se asegura de que (nombre del bebé) no coma alimentos azucarados como dulces, helados, pasteles o galletas.					
23d. Usted se asegura de que (nombre del bebé) no coma comida chatarra como papas fritas, Doritos y hojaldres de queso					

23e. Usted trata de hacer que (nombre del bebé) termine su comida					

How frequently do you engage in the following behaviors?	Never	Rarely	Half of the time	Most of the time	Always
24a. If (name of child) seems full, you encourage him/her to finish his/her food anyway					
24b. You try to get (name of child) to finish his/her breastmilk or formula					
24c. You try to get (name of child) to eat even if she/he is not hungry					
24d. If (name of child) will not try a new food that you give him/her, you will work hard to have him/her try it during that meal					
24e. You praise (name of child) after each bite to encourage him/her to finish his/her food					
24f. You give (name of child) cereal in the bottle					
24g. When (name of child) cries, you immediately feed him/her					
24h. You carefully control how much (name of child) eats					
24i. You are very careful not to feed (name of child) too much					
24j. You let (name of child) eat fast food					
24k. You let (name of child) decide how much to eat					
24l. You pay attention when (name of child) seems to be telling you that s/he is full or hungry					
24m. You allow (name of child) to eat when s/he is hungry					
24n. You talk to (name of child) to encourage him/her to drink his/her formula/breastmilk					

¿Con qué frecuencia usted participa en los siguientes comportamientos?	Nunca	Raramente	Mitad de las veces	La mayoría de las veces	Siempre
24a. Si (nombre del bebé) parece estar lleno/a, lo/a anima a terminar su comida de todos modos					
24b. Usted trata de hacer que (bebé) termine la leche materna o la fórmula					
24c. Usted trata de hacer que (bebé) coma aunque no tenga hambre					
24d. Si (nombre del bebé) no prueba un nuevo alimento que le da, usted trabaja duro para que lo pruebe durante esa comida					
24e. Usted le celebra a (nombre de bebé) después de cada mordida para animarlo/a que él/ella termine la comida					
24f. Usted le da cereales a (nombre del bebé) en el biberon					
24g. Cuando (nombre del bebé) llora, usted de inmediatamente le da de comer a él/ella					
24h. Usted cuidadosamente controla la cantidad que (nombre del bebé) come					
24i. Usted tiene mucho cuidado de no alimentar a (nombre del bebé) demasiado					
24j. Usted deja a (nombre del bebé) comer comida rápida					
24k. Usted deja que (nombre del bebé) decida cuánto comer					
24l. Usted presta atención cuando (nombre del bebé) parece estar diciéndole que está lleno/a o cuando tiene hambre					
24m. Usted deja que (nombre del bebé) coma cuando tiene hambre					
24n. Usted habla con (nombre del bebé) para animarla/lo a tomar su fórmula/leche materna					

	Yes	No
25a. Has your baby eaten or tried to eat <u>any solid foods any time</u> since birth?		

	Age in months	Age in weeks
25b. How old was your baby when they first tried solid foods?		

	Yes	No
26. In the past two weeks have you fed your baby either formula or pumped breast milk with a bottle?		

How often have you added each of the following items to your baby's bottle of formula or pumped (or expressed) breast milk in the <u>past 2 weeks?</u>

	Never	Only rarely	Every few days	About once a day	At most feedings	Every feeding
27a. Vitamins or minerals						
27b. Baby cereal						
27c. Sweetener, such as sugar						
27d. Other.<br>27e. Please specify:						

	Sí	No
25a. ¿Su bebé ha comido o intentado comer alimentos sólidos en algún momento desde que nació?		

	Edad en meses	Edad en semanas
25b. ¿Que edad tenía su bebé cuando probó alimentos sólidos por primera vez?		

	Sí	No
26. En las últimas dos semanas, ¿ha alimentado a su bebé con fórmula o con leche materna extraída con un biberón?		

¿Con qué frecuencia ha agregado cada uno de los siguientes artículos al biberón de fórmula de su bebé o la leche materna (o extraída) en las últimas 2 semanas?

	Nunca	Solo raramente	Cada pocos días	Aproximadamente una vez al día	En la mayoría de las comidas	Cada alimentación
27a. Vitaminas o minerales						
27b. Cereales para bebés						
27c. Endulzante, como azucar						
27d. Otro artículo.<br>27e. Por favor especifique:						

How frequently do you engage in the following behaviors?	Never	Rarely	Half of the time	Most of the time	Always
28a. You talk to (name of child) to encourage him/her to eat					
28b. You show (name of child) how to eat by taking a bite yourself or pretending to take a bite					
28c. You will retry offering new foods if they are rejected at first					
28d. You allow (name of child) to watch TV while eating if s/he wants to do so					
28e. You allow (name of child) to eat fast food if s/he wants to do so					
28f. You allow (name of child) to drink sugared drinks/soda if s/he wants to do so					
28g. You allow (name of child) to eat desserts/sweets if s/he wants to do so					
28h. You allow (name of child) to watch TV while eating to make sure s/he gets enough					
28i. You allow (name of child) to eat fast food to make sure s/he gets enough					
28j. You allow (name of child) to drink sugared drinks/soda to make sure s/he gets enough					
28k. You allow (name of child) to eat desserts/ sweets to make sure s/he gets enough					
28l. You allow (name of child) to watch TV while eating to keep him/her from crying					
28m. You allow (name of child) to eat fast food to keep him/her from crying					
28n. You allow (name of child) to drink sugared drinks/soda to keep him/her from crying					
28o. You allow (name of child) to eat desserts/ sweets to keep him/her from crying					

¿Con qué frecuencia usted participa en los siguientes comportamientos?	Nunca	Raramente	Mitad de las veces	La mayoría de las veces	Siempre
28a. Usted habla con (nombre del bebé) para animarla/o a comer					
28b. Usted le muestra a (nombre del bebé) cómo comer mordiendo o pretendiendo					
28c. Usted vuelve a ofrecer nuevos alimentos si se rechazan al principio					
28d. Usted permite que (nombre del bebé) mire la televisión mientras come si lo desea					
28e. Usted permite que (nombre del bebé) coma comida rápida si lo desea					
28f. Usted permite que (nombre del bebé) tome bebidas azucaradas/refrescos si lo desea					
28g. Usted permite que (nombre del bebé) coma postres/dulces si lo desea					
28h. Usted permite que (nombre del bebé) vea la televisión mientras come para asegurarse de que coma lo suficiente					
28i. Usted permite que (nombre del bebé) coma comida rápida para asegurarse de que coma lo suficiente					
28j. Usted permite que (nombre del bebé) tome bebidas azucaradas/refrescos para asegurarse de que coma lo suficiente					
28k. Usted permite que (nombre del bebé) coma postres/dulces para asegurarse que él/ella coma lo suficiente					
28l. Usted permite que (nombre del bebé) mire televisión mientras come para evitar que llore					
28m. Usted permite que (nombre del bebé) coma comida rápida para evitar que llore					
28n. Usted permite que (nombre del bebé) beba bebidas azucaradas/refrescos para evitar que llore					
28o. Usted permite que (nombre del bebé) coma postres/dulces para evitar que llore					

	At most bedtimes, including naps	At most night bedtimes, but not naps	At most naps, but not night bedtimes	Only occasionally at bedtimes, including naps	Never
29. During the past 2 weeks how often was your baby put to bed with a bottle of formula, breast milk, juice, juice drink, or any other kind of milk?					

	Yes	No
30. Do you encourage your infant to use a spoon or finger to feed themselves?		
31. Do you encourage your infant to drink from a cup?		

	Yes	No
32a. Does anyone besides you help prepare the infant's food and meals?		

How often do these people help prepare the infant's food and meals?	Never	Rarely	Half of the time	Most of the time	Always
32b. The baby's father					
32c. Your mother					
32d. Your mother-in-law					
32e. Other family member					
32f. Non-family member babysitter					
32g. Other person<br>32h. Please specify:					

	En la mayoría de las horas de dormir, incluyendo las siestas	En la mayoría del tiempo a la hora de dormir en la noche, pero no en las siestas	En la mayoría de las siestas, pero no en la hora de dormir en la noche	Solo a veces a la hora de dormir, incluyendo las siestas	Nunca
29. Durante las últimas 2 semanas, ¿con qué frecuencia su bebé fue acostado/a con un biberón de fórmula, leche, jugo, bebida de jugo o cualquier otro tipo de leche?					

	Sí	No
30. ¿Anima a su bebé a usar una cuchara o un dedo para alimentarse?		
31. ¿Anima a su bebé a beber de una taza?		

	Sí	No
32a. ¿Alguien además de usted ayuda a preparar las comidas del bebé?		

¿Con qué frecuencia ayudan estas personas a preparar la comida y las comidas del bebé?	Nunca	Raramente	La mitad de las veces	La mayoría de las veces	Siempre
32b. El padre del bebé					
32c. Su madre					
32d. Su suegra					
32e. Otro miembro de la familia					
32f. Niñera, no familiar					
32g. Otra persona.<br>32h. Por favor especifique:					

How frequently do you engage in the following behaviors?	Never	Rarely	Half of the time	Most of the time	Always
33a. You allow (name of child) to watch tv while eating to keep him/her happy					
33b. You allow (name of child) to eat fast food to keep him/her happy					
33c. You allow (name of child) to drink sugared drinks/soda to keep him/her happy					
33d. You allow (name of child) to eat desserts/sweets to keep him/her happy					
33e. You offer snacks to make sure (name of baby) is eating enough.					
33f. You offer (name of baby) snacks as a reward for good behavior.					
33g. You offer snacks to make (name of baby) feel better.					

	Yes	No
34a. Has your baby tried to eat or eaten any lumpy foods any time since birth? Lumpy foods are not pureed, but they are soft, chopped, ground, or mashed foods, such as mashed potatoes or mashed bananas.		

	Age in months	Age in weeks
34b. How old was your baby when they first tried the lumpy foods?		

¿Con qué frecuencia usted participa en los siguientes comportamientos?	Nunca	Raramente	Mitad de las veces	La mayoría de las veces	Siempre
33a. Usted permite que (nombre del bebé) mire televisión mientras come para mantener a él/ella feliz					
33b. Usted permite que (nombre del bebé) coma comida rápida para mantener a él/ella feliz					
33c. Usted permite que (nombre del bebé) beba bebidas azucaradas/refresco para mantener a él/ella feliz					
33d. Usted permite que (nombre del bebé) coma postres/dulces para mantener a él/ella feliz					
33e. Usted ofrece aperitivos para asegurarse de que (nombre del bebé) coma lo suficiente.					
33f. Usted ofrece aperitivos a (nombre del bebé) como recompensa por buen comportamiento.					
33g. Usted ofrece aperitivos para que (nombre del bebé) se sienta mejor.					

	Sí	No
34a. ¿Ha intentado su bebé comer algún alimento con grumos en algún momento desde su nacimiento? La comida grumosa no se hace como crema, pero esta ablandada, picada, molida, como puré de papas.		

	Edad en meses	Edad en semanas
34b. ¿Qué edad tenía su bebé cuando probó los alimentos con grumos por primera vez?		

MATERNAL CHARACTERISTICS

The next questions ask for background information about you.

	35a. Month	35b. Day	35c. Year
What is your birth date?			

	Feet	Inches
36. What was your height in feet and inches prior to becoming pregnant with (name of baby)?		

	Pre-pregnancy weight
37. What was your weight in pounds prior to becoming pregnant with (name of baby)?	lbs.

	Yes	No
38. When you were pregnant with (name of baby), did a doctor diagnose you as having gestational diabetes?		

	Yes	No
39. Are you covered by health insurance?		

	Private insurance	Public insurance (MediCal/Medicare)	Other. 40b. Please specify.
40a. What health insurance do you have?			

	Yes	No
40c. Have you or anyone in your household ever served in the U.S. Armed Forces, military Reserves, or National Guard?		

CARACTERÍSTICAS MATERNAS

Las siguientes preguntas solicitan información sobre usted.

	35a. Mes	35b. Día	35c. Año
¿Cuál es su fecha de nacimiento?			

	Pies	Pulgadas
36. ¿Cuál era su estatura en pies y pulgadas antes de quedar embarazada de (nombre del bebé)?		

	Peso antes del embarazo
37. ¿Cuál era su peso en libras antes de quedar embarazada de (Nombre del bebé)?	libras

	Sí	No
38. Cuando estaba embarazada de (nombre del bebé), ¿un médico le diagnosticó diabetes gestacional?		

	Sí	No
39. ¿Usted está cubierta por un seguro de salud?		

	Seguro privado	Seguro publico (MediCal/Medicare)	Otro.<br>40b. Por favor especifique.
40a. ¿Qué seguro de salud tienes?			

	Sí	No
40c. ¿Usted o alguien en su hogar ha servido en las Fuerzas Armadas de Estados Unidos, la Reserva militar o la Guardia Nacional?		

	Eighth grade or less	High school or General Equivalency Diploma	Vocational school or Associate's degree	Bachelor's or graduate degree
41. What is the highest level of education that you completed?				

	Employed full time	Employed part time	Not employed	Retired	Other. 42b. Please Specify:
42a. How would you describe your current employment status?					

	Yes	No
43a. Do you identify yourself as either Latina or Hispanic?		

	Fill in the blank.
43b. With what, if any, Latino or Hispanic subgroups, such as Mexican, Guatemalan, etc., do you identify?	

	Octavo grado o menos	Escuela secundaria o Diploma de equivalencia general	Escuela vocacional o grado de Asociado	Título de bachiller o posgrado
41. ¿Cuál es el nivel de educación más alto que completó?				

Instrucciones para el entrevistador: No necesitas definir "tiempo completo" y "tiempo parcial" a menos que el participante lo solicite. En ese caso, "tiempo parcial" es menos de 35 horas por semana.

	Empleado a tiempo completo	Empleado a tiempo parcial	No empleado	Jubilado	Otro.<br>42b. Por favor especifique:
42a. ¿Cómo describiría su situación laboral?					

Instrucciones para el entrevistador: si el participante no tiene claro cómo responder la siguiente pregunta, puede explicar que "Latina o hispana" se refiere a miembros de un grupo étnico que tiene sus raíces en 20 países que hablan español de América Latina y España.

	Si. Por favor especifique.	No
43a. ¿Usted se identifica como Latina o Hispana?		

	Completa el espacio en blanco.
43b. ¿Con qué subgrupos latinos o hispanos, si lo hay, como mexicano, guatemalteco, etc., se identifica?	

	White	Black or African American	American Indian or Alaska Native (Specify tribe)	Asian American, Native Hawaiian, or Pacific Islander	Other. 44b. Please specify:
44a. What is your race? Ok to choose more than one					

	United States	Mexico	Other country. 45b. Please specify:
45a. In what country were you born?			

	United States	Mexico	Other country.. 46b. Please specify:
46a. In what country was your mother born?			

	United States	Mexico	Other country. 47b. Please specify:
47a. In what country was your father born?			

	Yes	No
48. Are you a single parent?		

	Blanco	Negro o Afroamericano	Indio Americano o Nativo de Alaska (Especifique su tribu)	Asiático Americano, Nativo de Hawai o Isleño del Pacífico	Otro. 44b. Por favor, especifique
44a. ¿Cuál de las siguientes es su raza? Está bien elegir más que uno					

	Estados Unidos	México	Otro país. 45b. Por favor especifique:
45a. ¿En qué país nació usted?			

	Estados Unidos	México	Otro país. 46b. Por favor especifique:
46a. ¿En qué país nació su madre?			

	Estados Unidos	México	Otro país. 47b. Por favor especifique:
47a. ¿En qué país nació su padre?			

	Sí	No
48. ¿Es madre soltera?		

	Unmarried, not in a relationship	Unmarried, in a relationship with non-cohabiting partner	Unmarried, in a relationship with cohabiting partner	Married	Married but separated	Widowed
49a. What is your current relationship status?						

	Yes	No
49b. Is the relationship with the child's biological father?		

	Current smoker	Former smoker	Never a smoker
50. Are you a:			

The next set of questions asks about how you have been feeling lately.

Over the past two weeks, how often have you been bothered by any of the following problems?	Not at all	Several Days	More than half	Nearly every day
51a. Having little interest or pleasure in doing things				
51b. Feeling down, depressed, or hopeless				

	Soltera, en una relación	Soltera, en una relación con una pareja que no cohabita	Soltera, en una relación con una pareja que cohabita	Casada	Casada pero separada	Viuda
49a. ¿Cuál es su estado actual de relación?						

	Sí	No
49b. ¿Es la relación con el padre biológico del bebé?		

	Fumador actual	Ex fumador	Nunca un fumador
50. Es usted un...			

El siguiente conjunto de preguntas se refiere a cómo te has sentido últimamente.

En las últimas dos semanas, ¿con qué frecuencia le ha molestado alguno de los siguientes problemas?	Para nada	Varios días	Más de la mitad	Casi todos los días
51a. Poco interés o placer en hacer las cosas				
51b. Sentirse afligido, deprimido o desesperado				

The following items ask about anxiety and fear. Include all of your anxiety symptoms when answering these questions.

	No anxiety in the past week.	Infrequent anxiety. Felt anxious a few times.	Occasional anxiety. Felt anxious as much of the time as not. It was hard to relax.	Frequent anxiety. Felt anxious most of the time. It was very difficult to relax.	Constant anxiety. Felt anxious all of the time and never really relaxed.
52. In the past week, how often have you felt anxious?					

	Little or none: Anxiety was absent or barely noticeable.	Mild: Anxiety was at a low level. It was possible to relax when you tried. Physical symptoms were only slightly uncomfortable.	Moderate: Anxiety was distressing at times. It was hard to relax or concentrate, but you could do it if you tried. Physical symptoms were uncomfortable.	Severe: Anxiety was intense much of the time. It was very difficult to relax or focus on anything else. Physical symptoms were extremely uncomfortable.	Extreme: Anxiety was overwhelming. It was impossible to relax at all. Physical symptoms were unbearable.
53. In the past week, when you have felt anxious, how intense or severe was your anxiety?					

Los siguientes artículos preguntan sobre la ansiedad y el miedo. Incluya todos sus síntomas de ansiedad al responder estas preguntas.

	Sin ansiedad en la última semana.	Ansiedad infrecuente. Se sintió ansiosa algunas veces.	Ansiedad ocasional. Se sintió ansiosa la mayor parte del tiempo que no. Fue difícil relajarse.	Ansiedad frecuente. Se sintió ansiosa la mayor parte del tiempo. Fue muy difícil relajarse.	Ansiedad constante. Se sintió ansiosa todo el tiempo y nunca realmente relajado.
52. En la última semana, ¿con qué frecuencia se ha sentido ansiosa?					

	Poco o nada: La ansiedad estaba ausente o apenas se notaba.	Leve: La ansiedad estaba en un nivel bajo. Era posible relajarse cuando lo intentaba. Los síntomas físicos fueron solo un poco incómodos.	Moderado: La ansiedad era angustiante a veces. Fue difícil relajarse o concentrarse, pero podría hacerlo si lo intentara. Los síntomas físicos eran incómodos.	Grave: La ansiedad era intensa la mayor parte del tiempo. Fue muy difícil relajarse o concentrarse en otra cosa. Los síntomas físicos fueron extremadamente incómodos.	Extremo: La ansiedad era abrumadora. Era imposible relajarse en absoluto. Los síntomas físicos eran insoportables.
53. En la última semana, cuando se sintió ansiosa, ¿que intensa o severa fue su ansiedad?					

	None: You do not avoid places, situations, activities, or things because of fear.	Infrequent: You avoid something once in a while, but will usually face the situation or confront the object. Your lifestyle is not affected.	Occasional: You have some fear of certain situations, places, or objects, but it is still manageable. Your lifestyle has only changed in minor ways. You always or almost always avoid the things you fear when you are alone, but can handle them if someone comes with you.	Frequent: You have considerable fear and really try to avoid the things that frighten you. You have made significant changes in your lifestyle to avoid the object, situation, activity, or place.	All the time: Avoiding objects, situations, activities, or places has taken over your life. Your lifestyle has been extensively affected and you no longer do things that you used to enjoy.
54. In the past week, how often did you avoid situations, places, objects, or activities because of anxiety or fear?					

	Ninguno: No evita lugares, situaciones, actividades o cosas por miedo.	Infrecuente: Evita algo de vez en cuando, pero generalmente enfrenta la situación o enfrenta el objeto. Su estilo de vida no es afectada.	Ocasional: Tiene cierto miedo a ciertas situaciones, lugares u objetos, pero aún es manejable. Su estilo de vida solo ha cambiado de manera menor. Siempre o casi siempre evita las cosas que teme cuando está sola, pero puede manejarlas si alguien viene con usted.	Frecuente: Tiene un miedo considerable y realmente trata de evitar las cosas que la asustan. Hizo cambios significativos en su estilo de vida para evitar el objeto, la situación, la actividad o el lugar.	Todo el tiempo: Evitar objetos, situaciones, actividades o lugares se ha apoderado de su vida. Su estilo de vida se ha visto ampliamente afectado y ya no hace cosas que solía disfrutar.
54. En la última semana, ¿con qué frecuencia evitó situaciones, lugares, objetos o actividades debido a la ansiedad o el miedo?					

	None: No interference at work/home/school from anxiety.	Mild: Your anxiety has caused some interference at work/home/school. Things are more difficult, but everything that needs to be done is still getting done.	Moderate: Your anxiety definitely interferes with tasks. Most things are still getting done, but few things are being done as well as in the past.	Severe: Your anxiety has really changed your ability to get things done. Some tasks are still being done, but many things are not. Your performance has definitely suffered.	Extreme: Your anxiety has become incapacitating. You are unable to complete tasks and have had to leave school, have quit or been fired from your job, or have been unable to complete tasks at home and have faced consequences like bill collectors, eviction, etc.
55. In the past week, how much did your anxiety interfere with your ability to do the things you needed to do at work, at school, or at home?					

	Ninguno: Ninguna interferencia en el trabajo /hogar/ escuela debido a la ansiedad	Leve: Su ansiedad ha causado alguna interferencia en el trabajo/ hogar/ escuela. Las cosas son más difíciles, pero todo lo que hay que hacer todavía se está haciendo.	Moderado: Su ansiedad definitivamente interfiere con las tareas. La mayoría de las cosas todavía se están haciendo, pero se están haciendo pocas cosas tan bien como en el pasado.	Grave: Su ansiedad realmente ha cambiado su capacidad para hacer las cosas. Algunas tareas todavía se están haciendo, pero muchas cosas no. Su desempeño definitivamente ha sufrido.	Extremo: Su ansiedad se ha vuelto incapacitante. No puede completar las tareas y ha tenido que abandonar la escuela, renunciar o haber sido despedida de su trabajo, o no ha podido completar las tareas en casa y ha tenido consecuencias como cobrar facturas, desalojos, etc.
55. En la última semana, ¿Cuánto su ansiedad interfirió con su habilidad para hacer las cosas que necesitaba hacer en el trabajo, en la escuela o en casa?					

	None: Your anxiety doesn't affect your relationships.	Mild: Your anxiety slightly interferes with your relationships. Some of your friendships and other relationships have suffered, but, overall, your social life is still fulfilling.	Moderate: You have experienced some interference with your social life, but you still have a few close relationships. You don't spend as much time with others as in the past, but you still socialize sometimes.	Severe: Your friendships and other relationships have suffered a lot because of anxiety. You do not enjoy social activities. You socialize very little.	Extreme: Your anxiety has completely disrupted your social activities. All of your relationships have suffered or ended. Your family life is extremely strained.
56. In the past week, how much has anxiety interfered with your social life and relationships?					

HOUSEHOLD CHARACTERISTICS

The next questions ask about your household.

	Zip code
57. In what zip code do you live?	

	Rent	Own
58. Do you rent or own your current residence?		

	Ninguno: Su ansiedad no afecta sus relaciones.	Leve: Su ansiedad interfiere ligeramente con sus relaciones. Algunas de sus amistades y otras relaciones han sufrido, pero, en general, su vida social todavía se está cumpliendo.	Moderada: Ha experimentado alguna interferencia con su vida social, pero todavía tiene algunas relaciones cercanas. No pasó tanto tiempo con otros como en el pasado, pero todavía socializa a veces.	Grave: Sus amistades y otras relaciones han sufrido mucho debido a la ansiedad. No disfruta de las actividades sociales. Socializa muy poco.	Extremo: Su ansiedad ha interrumpido por completo sus actividades sociales. Todas sus relaciones han sufrido o han terminado. Su vida familiar es extremadamente tensa.
56. En la última semana, ¿Cuánto la ansiedad interferio con su vida social y relaciones?					

CARACTERÍSTICAS DEL HOGAR

Las siguientes preguntas son sobre su hogar.

	Código postal
57. ¿En qué código postal vive?	

	Renta	Dueño
58. ¿Renta o es dueño de su residencia?		

	Eighth grade or less	High school or GED	Vocational school or Associate's degree	Bachelor's or graduate degree
59. Thinking of all of the people who are living in your household, including yourself, what is the highest level of education that someone in your household has completed?				

60. During the past 3 months, what was the average total monthly income of your household before taxes? Your household includes you and anyone who lives with you <u>and</u> depends on the same income. Be sure to include income from all sources, such as salary and wages, child support, interest, public assistance and pensions. If you don't know your household's monthly income, you can answer in terms of average income per week or year.

Per month		Per week	Per year
Less than $417		Less than $97	Less than $5,000
$418 to $833		$97 to $192	$5,001 to $10,000
$834 to $1,250		$193 to $288	$10,001 to $15,000
$1,251 to $1,666		$289 to $384	$15,001 to $20,000
$1,667 to $2,083		$385 to $480	$20,001 to $25,000
$2,084 to $2,500		$481 to $576	$25,001 to $30,000
$2,501 to $2,916		$577 to $673	$30,001 to $35,000
$2,917 to $3,333		$674 to $769	$35,001 to $40,000
$3,334 to $3,750		$770 to $865	$40,001 to $45,000
$3,751 to $4,166		$866 to $961	$45,001 to $50,000
$4,167, to $4,583		$962 to $1,057	$50,001 to $55,000
$4,584 to $5,000		$1,058 to $1,153	$55,001 to $60,000
$5,001 to $5,416		$1,154 to $1,250	$60,001 to $65,000
$5,417 to $5,833		$1,251 to $1,346	$65,001 to $70,000
More than $5,833		More than $1,346	More than $70,000

	Octavo grado o menos	Escuela secundaria o Diploma de equivalencia general	Escuela vocacional o título de Asociado	Título de bachiller o posgrado
59. Pensando en todas las personas en su hogar, incluyendo usted, ¿cuál es el nivel más alto de educación que alguien en su hogar ha completado?				

Durante los últimos 3 meses, ¿cuál fue el ingreso mensual promedio total de su hogar antes de impuestos? Su hogar lo incluye a usted y a cualquier persona que viva con usted y depende del mismo ingreso. Asegúrese de incluir los ingresos de todas las fuentes, como sueldos y salarios, manutención de los hijos, intereses, asistencia pública y pensiones. Si no se sabe el ingreso mensual de su hogar, puede responder en términos de ingreso promedio por semana o año.

Por mes		Por semana	Por año
Menos de $417		Menos de $97	Menos de $5,000
$418 a $833		$97 a $192	$5,001 a $10,000
$834 a $1,250		$193 a $288	$10,001 a $15,000
$1,251 a $1,666		$289 a $384	$15,001 a $20,000
$1,667 a $2,083		$385 a $480	$2067 $25.000
$2,084 $ 2,500		$481 a $576	$25,001 a $30,000
$2,501 a $2,916		$577 a $673	$30,001 a $35,000
$2,917 a $3,333		$674 a $769	$35,001 a $40,000
$3,334 a $3,750		$770 a $865	$40,001 a $45,000
$3,751 a $4,166		$866 a $961	$45,001 a $50,000
$4,167 a $4,583		$962 a $1,057	$50,001 a $55,000
$4,584 a $5,000		$1,058 a $1,153	$55,001 a $60,000
$5,001 a $5,416		$1,154 a $1,250	$60,001 a $65,000
$5,417 a $5,833		$1,251 a $1,346	$65,001 a $70,000
Más de $5,833		Más de $1,346	Más de $70,000

	Number
61. During the past 3 months, how many adults, including yourself, were supported by this household income?	
62. During the past 3 months, how many children were supported by this household income?	

Here is a list of non-cash government benefits. Has your household received any support from these sources in the past 3 months?	Yes	No
63a. Food Stamps or SNAP		
63b. WIC		
63c. Housing voucher, such as for Section 8 housing		
63d. Child care subsidy		
63e. Other benefit.<br>63f. Please specify_______________		

	Often	Sometimes	Never
64. Within the past 6 months, how often did you worry whether your food would run out before you got money to buy more?			
65. Within the past 6 months, how often did the food you bought not last and you didn't have money to get more?			

We would now like to ask you some questions about your home. Remember there are no right or wrong answers.

To what extent are these statements like your home or not like your home?	Very much like your own home	Somewhat like your own home	A little bit like your own home	Not at all like your own home
66a. There is very little commotion in your home				
66b. You can usually find things when you need them				
66c. You almost always seem to be rushed				
66d. You are usually able to stay on top of things				

	Número
61. Durante los últimos 3 meses, ¿cuántos adultos, incluyéndose usted, fueron apoyados por los ingresos de este hogar?	
62. Durante los últimos 3 meses, ¿cuántos niños fueron sostenidos con este ingreso familiar?	

Aquí hay una lista de beneficios gubernamentales no monetarios. ¿Ha recibido su casa algún apoyo de estas fuentes en los últimos 3 meses?	Si	No
63a. Cupones de alimentos o SNAP		
63b. WIC		
63c. Vales de elección de vivienda, como la sección 8		
63c. Subsidio de cuidado infantil		
63d. Otro, Por favor especifique________________		

	Con frecuencia	A veces	Nunca
64. En los últimos 6 meses, ¿con qué frecuencia se preocupaba si su comida se acabara antes de obtener dinero para comprar más?			
65. En los últimos 6 meses, ¿con qué frecuencia la comida que compró no duró y no tuvo dinero para obtener más?			

Ahora nos gustaría hacerle algunas preguntas sobre su hogar. Recuerde que no estamos aquí para juzgarla ni buscamos respuestas correctas o incorrectas.

¿Estas declaraciones son como su hogar o no como su hogar?	Muy parecido a su propia casa	Algo parecido a su propia casa	Un poco como su propia casa	No como su propia casa
66a. Hay muy poca conmoción en su casa				
66b. Por lo general, ustedes pueden encontrar cosas cuando las necesitan				
66c. Ustedes casi siempre tienen prisa				
66d. Generalmente ustedes son capaces de estar al tanto de las cosas				

Are these statements like your home or not like your home?	Very much like your own home	Somewhat like your own home	A little bit like your own home	Not at all like your own home
67a. No matter how hard you try, you always seem to be running late				
67b. It's a real zoo in your home				
67c. At home you can talk to each other without being interrupted				
67d. There is often a fuss going on at your home				
67e. No matter what your family plans, it usually doesn't seem to work out				
67f. You can't hear yourself think in your home				
67g. You often get drawn into other people's arguments at home				
67h. Your home is a good place to relax				
67i. The telephone takes up a lot of time at your home				
67j. The atmosphere in your home is calm				
67k. First thing in the day, you have a regular routine at home				

ACCULTURATION

The next questions ask about your cultural identity and practices.

	Strongly Disagree	Somewhat Disagree	Somewhat Agree	Strongly Agree
68a. You think of yourself as being U.S. American.				
68b. You feel good about being U.S. American.				
68c. You feel that you are part of U.S. American culture.				
68d. You are proud of being U.S. American.				

¿Estas declaraciones son como su hogar o no como su hogar?	Muy parecido a su propia casa	Algo parecido a su propia casa	Un poco como su propia casa	No como su propia casa
67a. No importa cuánto lo intentan, ustedes siempre parecen llegar tarde				
67b. Es un relajo en su casa				
67c. En casa ustedes pueden hablar entre ustedes sin ser interrumpidos.				
67d. Con frecuencia hay un escándalo su nuestra casa				
67e. No importa cuál sea sus planes familiares, por lo general no parecen trabajar				
67f. Ustedes no pueden oírse pensar su casa				
67g. Con frecuencia se inmersa en discusiones de otras personas en el hogar				
67h. Su casa es un buen lugar para relajarse				
67i. El teléfono toma mucho tiempo en su casa				
67j. El ambiente en su hogar es tranquilo				
67k. Lo primero en el día, ustedes tienen una rutina regular en casa				

ACCULTURACIÓN

Las siguientes preguntas sobre sus identificaciones y comportamientos culturales.

	Muy en desacuerdo	Algo en desacuerdo	Algo de acuerdo	Muy de acuerdo
68a. Usted se considera estadounidense				
68b. Usted se siente bien de ser estadounidense				
68c. Usted siente que forma parte de la cultura estadounidense				
68d. Usted siente orgullo de ser estadounidense				

In an earlier question (#43a, 44a) you identified yourself as ______. Thinking of that group/ those groups, to what extent do you agree or disagree with the following statements?	Strongly Disagree	Somewhat Disagree	Somewhat Agree	Strongly Agree
69a. You think of yourself as being (group/s).				
69b. You feel good about being (group/s).				
69c. You feel that you are part of (group/s) culture.				
69d. You are proud of being (group/s).				

	Not at all	A little	Pretty Well	Extremely well, or like a native
70a. How well do you speak English in general?				
70b. How well do you understand English in general?				
70c. How well do you know popular U.S.-American newspapers and magazines?				
70d. How well do you know popular US-American actors and actresses?				
70e. How well do you know US-American History?				
70f. How well do you know US-American political leaders?				

En una pregunta anterior (# 43a, 44a) usted se identifico como _____. Pensando en ese grupo/ esos grupos, ¿en qué medida está de acuerdo o en desacuerdo con las siguientes declaraciones?	Muy en desacuerdo	Algo en desacuerdo	Algo de acuerdo	Muy de acuerdo
69a. Usted se considera (grupo/s).				
69b. Usted se siente bien de ser (grupo/s).				
69c. Usted siente que forma parte de la cultura (grupo/s).				
69d. Usted siente orgullo de ser (grupo/s).				

	Nada en absoluto	Un poco	Bastante bien, o como nativo	Muy bien
70a. ¿Qué tan bien habla inglés en general?				
70b. ¿Qué tan bien entiende el inglés en general?				
70c. ¿Qué tan bien conoce los periódicos y revistas populares de Estados Unidos?				
70d. ¿Qué tan bien conoce a actores y actrices populares estadounidenses?				
70e. ¿Qué tan bien conoce la historia estadounidense?				
70f. ¿Qué tan bien conoce a los líderes políticos estadounidenses?				

	Not at all	A little	Pretty Well	Extremely well, or like a native
71a. Do you speak a language other than English? How well do you speak that language in general?				
71b. How well do you understand that language in general?				
71c. How well do you know popular newspapers and magazines in (the country you or your family comes from)?				
71d. How well do you know popular actors and actresses from (the country you or your family comes from)?				
71e. How well do you know history of (the country you or your family comes from)?				
71f. How well do you know political leaders from (the country you or your family comes from)?				

	Nada en absoluto	Un poco	Bastante bien	Muy bien
71a. ¿Usted habla otro idioma que no sea inglés? ¿Qué tan bien usted habla ese idioma en general?				
71b. ¿Qué tan bien usted entiende ese idioma en general?				
71c. ¿Qué tan bien usted conoce los periódicos y revistas populares en (el país de donde viene usted o su familia)?				
71d. ¿Qué tan bien usted conoce a actores y actrices populares de (el país de donde viene usted o su familia)?				
71e. ¿Qué tan bien usted conoce la historia de (el país de donde viene usted o su familia)?				
71f. ¿Qué tan bien usted conoce a los líderes políticos de (el país de donde viene usted o su familia)?				
