# Supplementary material for: Grow well/Crecer bien: a protocol for research on infant feeding practices in low-income families
Source: BMC Public Health. 2020 Sep 21;20:1431. doi: 10.1186/s12889-020-09471-1 (PMC7503435; doi:10.1186/s12889-020-09471-1)
Supplement: Supplementary file 2 — Additional file 2: Appendix B. Feeding Diary Debriefing Interview. [file 12889_2020_9471_MOESM2_ESM.pdf]

Appendix B. FEEDING DIARY DEBRIEFING INTERVIEW

Diary Debriefing Interview Guide

Introduction. Today we will review the information you provided using Baby Connect. I'd like to talk about times when others helped you feed your baby during the 48 hours you used the app. This person would be involved in the care of your child on a regular basis and at least 3 hours or more a week. Second, I've selected some things you noted in the app that I'd like to know more about.

When thinking about this 48 hours of feeding your baby, tell us about times when you and another caregiver talked about or communicated about feeding your baby. For example, the discussion may have been about what, or how, or when to feed your baby. Please keep in mind that responses are entirely based on your experiences.

(1) Let's begin with the first day of feeding your baby. Thinking back to this day, are there feedings that really stick out in your mind that you'd like to talk about? Explain.

  a. Ok. Now I have some questions about some of the things you wrote down during the first day of feeding your baby. You wrote down _______________, can you tell me more about this.

(2) Let's move on to the second day of writing things down. Thinking back to this day, are there times that really stick out in your mind and you'd like to talk about? Explain.

  a. I have some questions about the second day of feeding your baby. You wrote down _______________, can you tell me more about this. Explain.

(3) Did you learn anything about feeding your baby during the time you wrote things down/used this app? Did you learn anything about how others were feeding your baby? Were you surprised by anything you learned?

(4) Has anything changed since using this app? Like what, when, or how you or another caregiver is feeding your baby? How so?

(5) Is there anything else you'd like to share about using the Baby Connect app? Anything you else about others involved in feeding your baby?

ENTREVISTA INFORMATIVA DEL DIARIO DE ALIMENTACIÓN

Guía de Entrevistas Informativa Sobre Diario

Introducción. Hoy revisaremos la información que proporcionó con Baby Connect. Me gustaría hablar de los momentos en que otros le ayudaron a alimentar a su bebé durante las 48 horas que utilizó la aplicación. Esta persona está involucrada en el cuidado de su bebé de manera regular y al menos 3 horas o más a la semana. En segundo lugar, he seleccionado algunas cosas que usted anotó en la aplicación que me gustaría saber un poco más.

Cuando piense en estas 48 horas de alimentar a su bebé, cuéntenos acerca de las ocasiones en que usted y otro cuidador hablaron o comunicaron acerca de alimentar a su bebé. Por ejemplo, la discusión puede haber sido acerca de qué, cómo, o cuándo alimentar a su bebé. Tenga en cuenta que las respuestas se basan completamente en sus experiencias.

(1) Comencemos con el primer día de alimentar a su bebé. Pensando en ese día, ¿hay alimentos que realmente sobresalgan en su mente de los que le gustaría hablar? Explique.

a. Bien. Ahora tengo algunas preguntas sobre algunas de las cosas que escribió durante el primer día de alimentar a su bebé. Usted escribió _______________, ¿puede decirme más sobre esto?

(2) Pasemos al segundo día de escribir cosas. Pensando en este día, ¿hay ocasiones que realmente sobresalgan en su mente y de las que le gustaría hablar? Explique.

a. Tengo algunas preguntas sobre el segundo día de alimentar a su bebé. Usted escribió _______________, ¿puede decirme más sobre esto? Explique.

(3) Pensando en estas 48 horas, ¿hubo veces que usted o el otro cuidador introdujeron nuevos alimentos o combinaciones de alimentos tales como:

- cereales de arroz o alimentos para bebés?
- añadir azúcar o miel al jugo o al agua?
- mantequilla de maní?
- ¿Carnes?
- ¿verduras como patatas o maíz?
- ¿Fruta?
- legumbres como frijoles?
- algo más?

¿Puede decirme un poco sobre por qué usted o el otro cuidador introdujeron estos alimentos?

¿Hubo momentos en los que no quería o decidió no introducir nuevos alimentos o retuvo la comida? En caso que si, explíquelo. [*La sonda* para entender si la madre u otros cuidadores estaban reteniendo los alimentos, por ejemplo, podría tener miedo o no estar listo para introducir ciertos alimentos.]

(4) ¿Aprendió algo sobre alimentar a su bebé durante el tiempo que escribió/uso esta aplicación? ¿Aprendió algo sobre cómo otros estaban alimentando a su bebé? ¿Le sorprendió algo que aprendió?

(5) ¿Ha cambiado algo desde el uso de esta aplicación? ¿Cómo qué, cuándo o cómo usted u otro cuidador están alimentando a su bebé? ¿Cómo?

(6) ¿Hay algo más que le gustaría compartir sobre el uso de la aplicación Baby Connect? ¿Algo más sobre otras personas involucradas en alimentar a su bebé?
