# Supplementary material for: Grow well/Crecer bien: a protocol for research on infant feeding practices in low-income families
Source: BMC Public Health. 2020 Sep 21;20:1431. doi: 10.1186/s12889-020-09471-1 (PMC7503435; doi:10.1186/s12889-020-09471-1)
Supplement: Supplementary file 3 — Additional file 3: Appendix C. Dyadic Interview Guide. [file 12889_2020_9471_MOESM3_ESM.pdf]

Appendix C. DYADIC INTERVIEW GUIDE

Introduction: I'd like to talk to both of you about your experiences of taking care of [____]over the 48-hour feeding diary period. We will talk about your feeding practices and how they might be similar or different. I'd also like to talk about growth and weight gain among infants and risk for childhood obesity. This discussion is very informal. Please keep in mind that there are no right or wrong answers.

Questions:

1. How are you all related or connected to each other?

2. Let's start by talking about your experiences of caring for [insert infant's name]. How would you describe your roles? What is expected of you? What do you expect of each other?

3. Let's talk about feeding the baby. From the feeding diary, we know that both of you are involved in feeding the infant baby. Can you explain how this works

4. We know sometimes caregivers have different ideas about what to feed infants. I have a few examples from the feeding diary where you made different feeding decisions. I'd like to spend some time talking about these to understand them better. Let's talk about these two feeding events when [_____________]. Can you explain how you might be thinking differently here? And what about this feeding event? Can you explain the difference here again?

   Probe: What kind of things were you thinking when you decided to feed the baby this? What does it mean to you when you feed the baby this?

5. Now that we've reviewed several differences in feeding decisions over the 48-hour feeding diary, let's take some time to talk about what you do differently and what you do similarly in terms of feeding the infant child. And what these feeding behaviors mean.

6. We know that feeding is not just about your baby getting nutrients, it's also about learning. For example, feeding a baby is also about teaching them how to eat (chew, hold a spoon) and what to eat (healthy foods). What does feeding mean to you all? And are there any examples from the 48-hour feeding diary that can help explain what you mean?

7. Thinking about some of the differences in how you fed the infant child, how do you typically deal with these differences?

GUÍA DE ENTREVISTA DIÁDICA

Introducción: Me gustaría hablar con los/as dos sobre sus experiencias de cuidar de [_____] durante el período de 48 horas del diario de alimentación. Hablaremos sobre sus prácticas de alimentación y cómo podrían ser similares o diferentes. También me gustaría hablar sobre el crecimiento y el aumento de peso entre los bebés y el riesgo de obesidad infantil. Esta discusión es muy informal. Por favor, tenga en cuenta que no hay respuestas correctas o incorrectas.

Preguntas:

1. Comencemos hablando de sus experiencias de cuidar [incluir nombre del bebé]. ¿Cómo describiría sus roles? ¿Qué expectativas se espera de usted? ¿Qué esperan el uno del otro?

2. Hablemos de alimentar al/a bebé. Del diario de alimentación, sabemos que ambos/as están involucrados/as en la alimentación del bebé. ¿Puede explicar cómo funciona esto?

3. Sabemos que a veces los cuidadores tienen ideas diferentes sobre qué alimentar a los bebés. Tengo algunos ejemplos del diario de alimentación donde tomaron diferentes decisiones de alimentación. Me gustaría pasar algún tiempo hablando de esto para entenderlos mejor. Hablemos de estos dos eventos de alimentación cuando [_____]. ¿Puede explicar cómo podrían estar pensando diferente aquí? ¿Y qué hay de este evento de alimentación? ¿Puede explicar la diferencia aquí otra vez?

Sonda: ¿Qué tipo de cosas estaba pensando cuando decidió alimentar al bebé así? ¿Qué significa para usted cuando alimenta al bebé con eso?

4. Ahora que hemos revisado varias diferencias en las decisiones de alimentación durante el diario de alimentación de 48 horas, tomemos un tiempo para hablar sobre lo que hacen de manera diferente y lo que hacen de manera similar en términos de alimentar al niño/a. Y lo que significan estos comportamientos de alimentación.

5. Sabemos que la alimentación no se trata sólo de que su bebé obtenga nutrientes, sino también se trata de aprender. Por ejemplo, alimentar a un bebé también consiste en enseñarle a comer (masticar, sostener una cuchara) y qué comer (alimentos saludables). ¿Qué significa la alimentación para todos ustedes? ¿Hay algún ejemplo del diario de alimentación de 48 horas que pueda ayudar a explicar lo que quiere decir?

6. Pensando en algunas de las diferencias en cómo alimentaron al niño/a, ¿cómo suelen lidiar con estas diferencias?

8. Now’s let’s talk about baby’s growth and size in the first years of life. You will do this task separately and then we will talk about it. Take a look at these infant body images. First, select the infant size that you think

is typical. Second, pick the healthy infant size. Third, select the preferred or ideal infant size. Last, select the size of your infant.

Probe: Why did you select these? I notice some differences in what you both selected, why might you have different ideas about infant body size?

Baby body scale for boys.

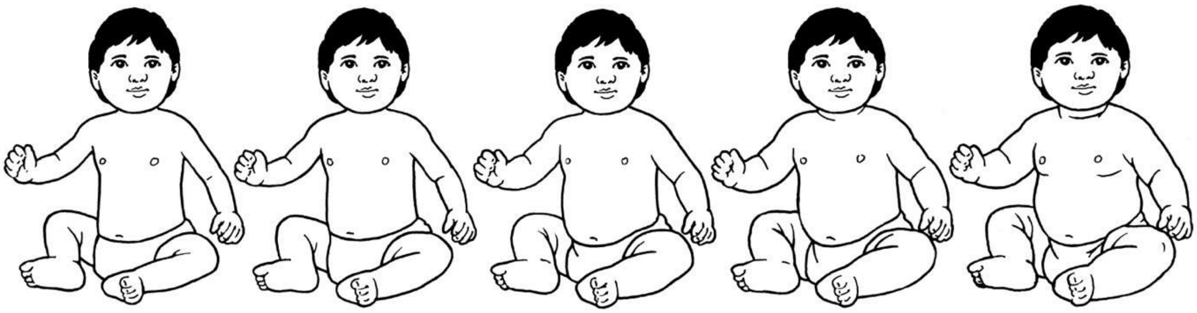

1 **2** **3** **4** **5**

Baby body scale for girls.

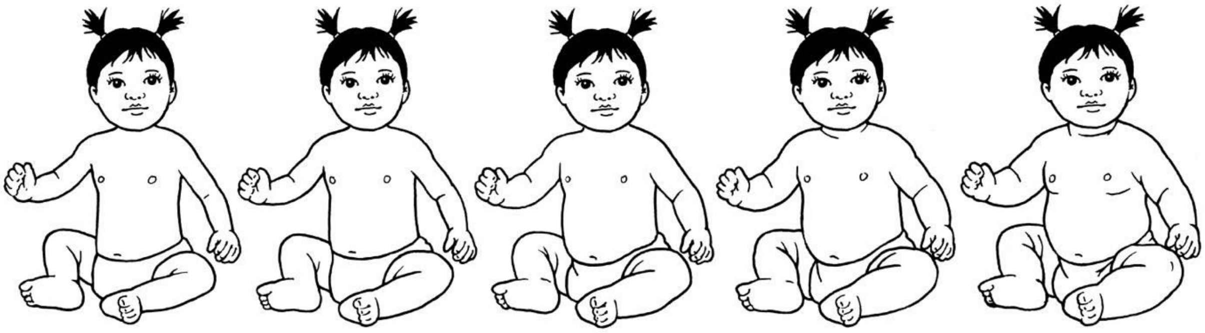

1 **2** **3** **4** **5**

7. Ahora hablemos del crecimiento y tamaño del bebé en los primeros años de vida. Usted hará esta tarea por separado y luego vamos a hablar de ello. Eche un vistazo a estas imágenes del cuerpo del bebé. En primer lugar, seleccione el tamaño del bebé que considere típico. En segundo lugar, escoja el tamaño saludable del bebé. En tercer lugar, seleccione el tamaño de bebé preferido o ideal. Por último, seleccione el tamaño de su bebé.

Sonda: ¿Por qué seleccionó estos? Noto algunas diferencias en lo que ambos/as seleccionaron, ¿por qué podrían tener ideas diferentes sobre el tamaño del cuerpo del bebé?

La escala de la silueta del cuerpo de niños

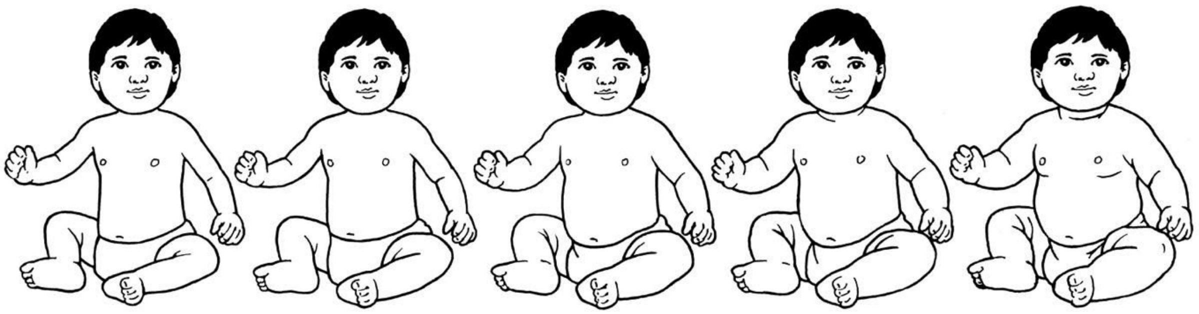

1 **2** **3** **4** **5**

La escala de la silueta del cuerpo de niñas

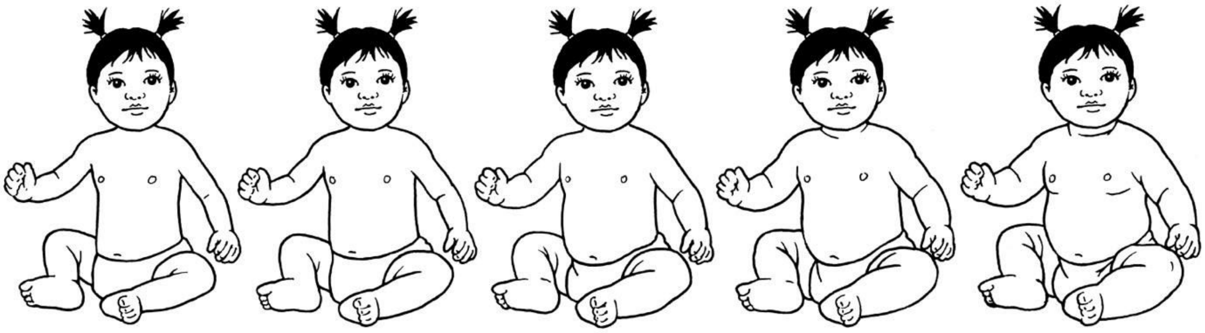

1 **2** **3** **4** **5**

9. Ok. Let's talk about concerns over too much or too little weight gain? Is this a concern in your family? Is there anything that you all have done that may lead to too much or too little weight gain for children in your family.

    Probe: What contributes to too little or too much weight gain?

10. Now we have some questions that we'd like you to answer using pen and paper about infant feeding practices and approaches to infant feeding [here only the other caregiver has to self-administer the Infant Feeding Practices and Styles Questionnaire].
