# Supplementary material for: Grow well/Crecer bien: a protocol for research on infant feeding practices in low-income families
Source: BMC Public Health. 2020 Sep 21;20:1431. doi: 10.1186/s12889-020-09471-1 (PMC7503435; doi:10.1186/s12889-020-09471-1)
Supplement: Supplementary file 4 — Additional file 4: Appendix D. Focus Group Guide. [file 12889_2020_9471_MOESM4_ESM.pdf]

Appendix D. FOCUS GROUP GUIDE

Introduction. Today we'd like to talk about healthy infant growth and feeding. To begin our conversation, we will share what we learned from families enrolled in EHS and related programs. We will also share information on some nutrition education programs for infants. Then we will get your thoughts on how we can use what we learned to improve nutrition education for families enrolled in EHS with infant children.

Overview of research

Over the past several years, we have spoken with mothers, collected information on feeding their infant children, measured their infant's weight and length, as well as talked with other caregivers helping with childcare. This is what we've done thus far:

<u>We've conducted interviews with mothers</u>: About 300 mothers and their children participated in surveys during a two-year period. The children were about 2 months of age when they began the study and 24 months at the end of the study. Mothers completed 4 surveys during the two years. We asked mothers questions about what they thought about (attitudes), when feeding their child. We also asked about what they did when feeding their child. And, we measured their children's weight and length. We did this, four times. When the child was 2 months, 6 months, 12 months and 24 months. This is what we learned:

[insert key bullet points from the longitudinal cohort study]

Feedback on findings:

a) We'd like to get your thoughts on this information.
    a) First, does this sound right?
    b) Second, how can we use this information to help improve programs for obesity prevention for children? What can we incorporate and why?

<u>We also conducted feeding diaries and interviews with mothers and trusted other caregivers</u>: We asked 36 of these mothers to record feeding their child for 48 hours. Mothers did this when their child was 2 and 6 months of age. We asked mothers to pay attention to what they fed their child, when, why, and if anyone else fed or helped feed their child. We also asked mothers to tell us about any challenges or conflicts they experienced with feeding their child. After reviewing this information, we sat down with mothers and another caregiver. Someone who they chose. Someone who also fed or helped feed their child. During the interview with the mother and other caregiver, we asked them to talk about challenges, conflicts, and disagreements they had with feeding the child they care for. This is what we learned:

[insert key bullet points from feeding diaries and dyadic interviews]

Feedback on findings:

b) We'd like to get your thoughts on this information.
    c) First, does this sound right?
    d) Second, is there a way for us to use this information to help improve programs for obesity prevention for children? What can we incorporate and why?

Questions.

We'd like to get your thoughts on how we can add the information we just discussed into existing Early Head Start nutrition programming.

1. First, tell us about what you've learned about nutrition through EHS. How has that been helpful?
   [or for staff: Tell us about the services you provide or information you share related to nutrition with EHS families.

2. We learned that EHS programs [here describe programs]. How can we build on this model or program?

   a. How can we include information on how to feed infants/babies?
   b. What information should we include?
   c. What about how mothers' think or believe about how babies should be fed?
   d. What about other caregivers' involvement in feeding? How should we think about this?

3. Thinking about the ideas we just discussed, how would this programing work?
   a. Who would lead the program?
   b. How would information be shared (e.g., video, talking)? What types of materials would people like?
   c. Who would participate in the program?
   d. Where would the program be held?
   e. How would we make sure that it includes things that are unique to people's cultures?
   f. Would there be anything that would make it hard for mothers and families to use this programing? How often and for how long should the programming be shared with mothers/families?
